# Rank charged system search algorithm for optimization and operations research

**DOI:** 10.1038/s41598-025-22956-6

**Published:** 2026-01-06

**Authors:** Mohamad Hosein Rabiei, Elnaz Eilbeigi, Siamak Talatahari, Mohammadtaghi Alami, Fang Chen, Amir H. Gandomi

**Affiliations:** 1 Department of Civil Engineering, University of Tabriz, Tabriz, Iran; 2 Department of Biomedical Engineering, ET.C., Islamic Azad University, Tehran, Iran; 3 Data Science Institute, Faculty of Engineering & Information Technology, University of Technology, Ultimo, 2007 Australia; 4 School of Computing, Macquarie University, Sydney, Australia; 5 Department of Computer Science, Khazar University, Baku, Azerbaijan; 6 University Research and Innovation Center (EKIK), Óbuda University, Budapest, 1034 Hungary

**Keywords:** Charged system search, Rank-based method, Optimization, Meta-heuristic algorithms, Data clustering, Reservoir operation optimization, Civil engineering, Applied mathematics, Computational science, Computer science

## Abstract

In this paper, we introduce CSSRank, an improved version of the charged system search (CSS) algorithm, designed to address complex optimization problems more efficiently. CSSRank integrates a rank-based reduction selection strategy to enhance exploitation by progressively reducing the number of charged particles used in electric force calculations. To further balance exploration and exploitation, a ranking-based mutation strategy is incorporated, promoting diversity in early iterations and precision in later stages. We evaluated CSSRank on a set of standard benchmark functions and compared its performance with the original CSS algorithm. In addition, CSSRank was tested on two major benchmark suites, CEC 2014 and CEC 2024, and compared against a wide range of state-of-the-art metaheuristic algorithms. The results show that CSSRank outperforms many existing methods on CEC 2014 and performs competitively and close to the best-performing algorithms on CEC 2024, demonstrating both robustness and scalability. For real-world applications, CSSRank was applied to six UCI clustering datasets, where it consistently achieved higher clustering accuracy and more reliable objective values than baseline methods. It was also tested on three complex reservoir operation optimization problems, yielding superior engineering solutions with high reliability, and contributing to improvements in operational cost and resource efficiency. These results confirm the effectiveness, versatility, and reliability of CSSRank across both theoretical and practical optimization tasks, positioning it as a strong candidate for solving complex problems in optimization and operations research.

## Introduction

Among the latest generation of optimization methods, meta-heuristic algorithms stand out for their ability to effectively tackle problems characterized by a large number of decision variables and diverse local solutions. These methods leverage stochastic principles inspired by natural phenomena. Moreover, they typically operate independently of initial starting points and do not rely on gradient information for the functions involved. In recent years, a multitude of meta-heuristic optimization algorithms has emerged demonstrating successful applications across various complex computational problems, such as mathematical problems^1–3^, engineering problems^3–6^, pattern recognition^7,8^, neural network learning^9–11^, image processing^12–14^, filter modeling^15,16^, data clustering problems^17,18^, as many others. References to various applications of meta-heuristic algorithms, along with their hybrid and modified versions for solving optimization problems, can be found in^19–22^.

The charged system search (CSS), a meta-heuristic algorithm, was introduced in 2010 by Kaveh and Talatahari^23^. Various engineering and mathematical optimization problems have been solved using this algorithm^24,25^. According to CSS, the governing equations of motion from Newtonian mechanics and Coulomb and Gauss’s laws of electrostatics govern the algorithm^23^. CSS optimizes each charged particle’s position (CP) by determining its resultant force and then moving it using Newton’s laws of motion. The algorithm is directed toward optimal solutions by these successive movements of CPs.

While CSS has shown promise, it suffers from two key limitations in practice: (1) its reliance on full population interactions results in high computational cost, especially in large-scale problems, and (2) its search dynamics can be overly exploitative, causing premature convergence and stagnation in multimodal landscapes. These drawbacks limit its applicability in real-world scenarios such as reservoir operation optimization or large-scale data clustering, where both scalability and robustness are critical. Therefore, there is a need for a more efficient and flexible variant of CSS that balances exploration and exploitation while reducing computational burden.

In this study, our objective is to enhance the convergence process of CSS by introducing new features. To achieve this goal, we developed the rank-charged system search (CSSRank). CSSRank incorporates several well-known selection methods to minimize the number of CPs involved in calculating the resultant electrical force. This rank-based reduction not only reduces computation but also strengthens the algorithm’s focus on promising regions of the search space. Additionally, a mutation strategy introduces stochastic diversity to escape local optima and improve global search capability. A summary of the contributions of this work is as:

- **Introduction of CSSRank:** We propose CSSRank, an enhanced version of the population-based charged system search (CSS) algorithm, designed to address global optimization problems.
- **Incorporation of Rank-Based Reduction Selection**: CSSRank integrates a rank-based reduction selection strategy to enhance the algorithm’s exploitative capability, resulting in a reduction in the number of charged particles involved in calculating the resultant electric force as iterations progress.
- **Implementation of Mutation Strategy**: A mutation strategy is employed in CSSRank to prevent trapping in local optimal solutions, ensuring a balanced exploration-exploitation trade-off.
- **Evaluation Across Benchmark Functions and Application to Real Datasets and Reservoir Operation Optimization**: We optimize a variety of challenging benchmark functions using CSSRank and compare the results with those obtained using CSS and other meta-heuristic algorithms, demonstrating the robustness and efficiency of CSSRank. Also, we evaluate CSSRank using six real datasets from the UCI machine learning laboratory as well as three difficult reservoir operation optimization (ROO) problems, demonstrating its effectiveness in clustering data compared to alternative algorithms.

The remainder of this paper is organized as follows. Section "Literature review" provides a literature review of previous works, while a review of the standard CSS is briefly presented in Section "A review of the standard CSS algorithm". Section "Rank-based charged system search algorithm" describes the methodology of the proposed method. The application of CSSRank to different numerical examples to examine its efficiency is discussed in Section "Mathematical numerical experiments", followed by a comparison of the simulation results of CEC examples with those of the standard CSS and other meta-heuristic algorithms from the literature in Section "Application of CSSRank to data clustering". In Section "Application of CSSRank to optimal operation of multi-reservoir system", Reservoir Operation Optimization problems are defined and solved using the developed method. Finally, Section "Discussion and conclusion" concludes with a few remarks.

## Literature review

### Optimization methods

Optimization, the process of finding the best solution among a set of feasible alternatives, lies at the heart of numerous scientific and engineering disciplines. Over the years, a plethora of optimization algorithms have been developed, each offering unique strategies for solving complex problems across diverse domains. In this sub-section, we provide a review of state-of-the-art optimization methods, encompassing a broad spectrum of metaheuristic algorithms.

The landscape of optimization methods is vast and continually evolving, driven by the quest for more efficient and effective problem-solving techniques. From classical approaches rooted in mathematical programming to nature-inspired metaheuristics inspired by biological and physical phenomena, the arsenal of optimization algorithms has expanded significantly, catering to the diverse needs of researchers and practitioners alike.

Our review encompasses a curated selection of over 50 optimization algorithms, spanning decades of research and innovation. These algorithms represent a rich tapestry of methodologies, ranging from traditional techniques like Genetic Algorithms (GA) and Simulated Annealing (SA) to cutting-edge approaches such as Grasshopper Optimization Algorithm (GOA) and Ebola Optimization Search Algorithm (EOSA). Each algorithm offers distinct advantages and trade-offs, making them suitable for different problem domains and scenarios. Table 1 lists these algorithms.

**Table 1:** Overview of some meta-heuristic algorithms.

**Title**	**Description**	**Reference**	**Principle**
Charged system search (CSS)	Integrates principles from electrostatics and Newtonian mechanics to optimize solutions through the interaction of charged particles.	^23^	Physics
Whale optimization algorithm (WOA)	mimics the hunting behavior of humpback whales in search of prey, utilizing encircling, bubble-net, and spiral dynamics to optimize solutions.	^46^	Swarm
Salp swarm algorithm (SSA)	is based on the collective movement of salps in the ocean, optimizing solutions through swarm intelligence and movement dynamics.	^47^	Swarm
Flower pollination algorithm (FPA)	simulates the pollination process of flowering plants to optimize solutions, with flowers exchanging information to find optimal solutions.	^48^	Nature
Krill herd algorithm (KHA)	models the swarming behavior of krill in search of optimal solutions, with krill adjusting movement based on attraction, repulsion, and randomization.	^49^	Swarm
Moth flame optimization (MFO)	simulates the navigation behavior of moths towards a light source, optimizing solutions by attraction and avoidance of light sources.	^50^	Nature
Artificial bee colony (ABC)	optimizes solutions by simulating the foraging behavior of honeybee colonies, utilizing employed, onlooker, and scout bees to explore the solution space	^51^	Swarm
Equilibrium optimizer (EO)	simulates the equilibrium-seeking behavior of living organisms, optimizing solutions through balance and stability.	^52^	Physics
Grasshopper optimization algorithm (GOA)	mimics the swarming behavior of grasshoppers to optimize solutions, with grasshoppers adjusting movement based on attraction and repulsion forces.	^53^	Swarm
Arithmetic optimization algorithm (AOA)	utilizes arithmetic operations to generate new solutions, optimizing solutions through iterative refinement and adjustment.	^54^	Physics
Ebola optimization search algorithm (EOSA)	inspired by the spread of the Ebola virus to optimize solutions by mimicking the infection process.	^55^	Biology
Reptile search algorithm (RSA)	mimics the hunting behavior of reptiles to optimize solutions through stealth, patience, and strategic movements.	^56^	Biology
Dwarf mongoose optimization algorithm (DMOA)	inspired by the foraging behavior of dwarf mongooses, optimizing solutions through cooperative hunting and strategic planning.	^57^	Biology
Prairie dog optimization algorithm (PDOA)	inspired by the social behavior of prairie dogs, optimizing solutions through communication, cooperation, and vigilance.	^58^	Nature
Intelligent water drops (IWD)	mimics the movement of water drops in search of optimal paths, optimizing solutions through erosion, sedimentation, and accumulation.	^59^	Physics
Teaching-learning based optimization (TLBO)	simulates the teaching and learning process in classrooms, optimizing solutions through knowledge exchange and collaboration.	^60^	Society
Jaya algorithm (JA)	based on the optimization of a solution population by constantly improving and discarding inferior solutions, mimicking the relentless pursuit of excellence.	^61^	Mathematics
Eagle strategy (ES)	mimics the hunting behavior of eagles, optimizing solutions through keen observation, precise targeting, and rapid execution.	^62^	Nature
Imperialist competitive algorithm (ICA)	simulates the rise and fall of empires, optimizing solutions through competition, collaboration, and expansion.	^63^	Society
Bee algorithm (BA)	simulates the foraging behavior of bees, optimizing solutions through communication, collaboration, and adaptation.	^64^	Swarm
Virus optimization algorithm (VOA)	inspired by the spread and mutation of viruses, optimizing solutions through infection, mutation, and evolution.	^65^	Biology
Moth search algorithm (MSA)	inspired by the navigation behavior of moths towards light sources, optimizing solutions through attraction and adaptation.	^66^	Nature
Camel herds algorithm (CHA)	mimics the adaptive behavior of camels in arid environments, optimizing solutions through endurance, resilience, and resourcefulness.	^67^	Nature
Butterfly optimization algorithm (BOA)	inspired by the foraging behavior of butterflies, optimizing solutions through exploration, exploitation, and adaptation.	^68^	Swarm
Earthworm optimization algorithm (EOA)	simulates the burrowing and feeding behavior of earthworms, optimizing solutions through exploration, exploitation, and adaptation.	^69^	Nature
Jellyfish optimization algorithm (JOA)	inspired by the movement and propagation patterns of jellyfish, optimizing solutions through synchronization, pulsation, and expansion.	^70^	Swarm
Pigeon-inspired optimization (PIO)	mimics the navigation and homing behavior of pigeons, optimizing solutions through navigation, communication, and memory.	^71^	Nature
Glowworm swarm optimization (GSO)	inspired by the collective behavior of glowworms in attracting mates, optimizing solutions through bioluminescent signaling, spatial awareness, and clustering.	^72^	Swarm
Slime mould algorithm (SMA)	inspired by the growth and foraging behavior of slime molds, optimizing solutions through exploration, exploitation, and decentralized coordination.	^73^	Biology
Fruit fly optimization algorithm (FOA)	mimics the foraging behavior of fruit flies in locating food sources, optimizing solutions through exploration, exploitation, and adaptation.	^74^	Swarm
Worm optimization algorithm (WOA)	inspired by the burrowing and feeding behavior of worms, optimizing solutions through tunneling, navigation, and resource allocation.	^75^	Swarm
Sandpiper optimization algorithm (SOA)	mimics the flocking behavior of sandpipers in migrating and foraging, optimizing solutions through coordination, communication, and collective decision-making.	^76^	Biology
Sine cosine algorithm (SCA)	inspired by the sine and cosine functions, optimizing solutions through periodic oscillations and exploration of solution space.	^77^	Mathematics
Squirrel search algorithm (SSA)	mimics the foraging behavior of squirrels in locating and storing food, optimizing solutions through navigation, memory, and resource management.	^78^	Biology
Red fox optimization (RFO)	mimics the hunting and survival strategies of red foxes, optimizing solutions through adaptability, cunning, and efficient resource utilization.	^79^	Nature
Shuffled frog leaping algorithm (SFLA)	inspired by the group foraging behavior of frogs, optimizing solutions through individual exploration and social interaction within groups.	^80^	Swarm
Cheetah algorithm (CA)	mimics the hunting behavior of cheetahs, optimizing solutions through speed, agility, and pursuit of prey.	^81^	Nature
Chaos game optimization (CGO)	Utilizes chaotic dynamics inspired by the chaos game to explore and optimize solution spaces efficiently.	^82^	Mathematics
Multi-objective artificial bee colony (MOABC)	developed a Multi-Objective MOABC algorithm for energy-efficient scheduling.	^83^	Hybrid
Genetic-artificial bee colony (GABC)	Integrates genetic operators into ABC to solve line balancing and AGV scheduling problems.	^84^	Hybrid
Hybrid pareto spider monkey optimization (HPSMO)	Extends SMO for multi-objective PCB flow shop scheduling using Pareto dominance.	^85^	Hybrid
Improved artificial fish swarm algorithm (IAFSA)	Enhances AFSA for human–machine collaborative disassembly line balancing.	^86^	Hybrid
Hybrid PSO for cellular scheduling (HPSO)	Applied PSO to optimize product scheduling in cellular manufacturing system.	^87^	Hybrid
Raccoon family optimization (RFO)	Nature-inspired optimizers applied to integrated planning and scheduling under resource constraints.	^88^	Hybrid
Hybrid moth flame optimization (HMFO)	Provided hybrid MFO algorithm for global optimization	^89^	Hybrid
Self-adaptive moth flame optimizer with crossover operator and fibonacci search (SAMFO-CO-FS)	Combined self-adaptive moth flame optimizer with crossover operator and fibonacci search strategy for COVID-19 CT image segmentation	^90^	Hybrid
Quadratic and lagrange interpolation-based butterfly optimization algorithm (QLBO)	Combined quadratic and lagrange interpolation-based butterfly optimization algorithm for numerical optimization and engineering design problems	^91^	Hybrid
Opposite learning -based moth flame optimization algorithm (OLMFO)	Combined moth flame optimization algorithm with modified dynamic opposite learning strategy	^92^	Hybrid

The selection of the Charged System Search (CSS) algorithm as the foundation for our research stems from several compelling reasons. Firstly, the aim of this paper is to introduce new features that can be utilized in optimization methods. Therefore, we sought an algorithm that not only provided a solid foundation for implementing novel features but also exhibited flexibility and adaptability in addressing various optimization challenges. CSS, with its basis in electrostatic principles and population-based approach, emerged as a promising candidate. Moreover, while CSS may not boast the same level of popularity as some well-established algorithms like Genetic Algorithms (GA) or Particle Swarm Optimization (PSO), its underlying principles hold promise for addressing specific challenges in optimization. By leveraging electrostatic forces among charged particles to guide the search process, CSS exhibits a degree of adaptability and robustness that make it well-suited for tackling complex optimization problems with diverse solution landscapes.

### Reservoir operation optimization problem

Reservoir operation optimization (ROO) poses a multifaceted challenge due to its dynamic nature. The complexity of this problem necessitates the use of high-performance algorithms for practical solutions. This complexity arises from the presence of a large number of variables and the high computational cost associated with evaluating the objectives. Additionally, the presence of uncertain environmental factors further complicates the optimization process, requiring robust and efficient algorithms to navigate the problem landscape effectively.

Various studies have been conducted to address the challenge of solving ROO problems. Sharif and Wardlaw^26^ investigated the potential of GA in a real multi-reservoir case. This case study was carried out in a continuous domain without discretization. When the complexity increased, the amount of discretization work intensified, resulting in more computation time. According to Cai et al.^27^, evolutionary methods have been applied to solve large-scale nonlinear reservoir management models in recent years. Kumar and Reddy^28^ compared the performances of ant colony optimization (ACO) and GA in the operation of the Hirakud reservoir in India with agricultural, hydropower, and flood control functions. The transmission of information was based on probabilistic transition rules, which improved the solution in a short amount of time at every interval. This is substantially useful for obtaining a solution, especially in long-term planning. Bozorg-Haddad et al.^29^ studied the capability of the honeybee mating optimization (HBMO) algorithm in solving different operation system problems in both continuous and discrete domains. Dariane and Sarani^30^ employed the intelligent water drops (IWD) algorithm and ACO in Iran’s Dez reservoir operation problem. A comparison of the results shows that the IWD algorithm found relatively better solutions and can overcome the computational time consumption deficiencies inherited in the ACO methods. Bozorg-Haddad et al.^31^ applied the bat algorithm (BA) to a real case Karoun-4 reservoir operation, to optimize the reservoir operation system.

## A review of the standard CSS algorithm

In the CSS algorithm^23^, Newtonian mechanics and Coulomb’s law of electrostatic force are integrated to address diverse science and engineering problems. Within the CSS algorithm, CPs are assumed as candidate solutions capable of exerting attractive electric forces on each other according to Coulomb’s law. The motion of each CP is determined by calculating the resultant forces acting on it and applying the kinematic equations. Additionally, the charge magnitude of each CP is determined based on the value of the objective function^23^.

CPs have a uniform charge density and are considered charged spheres with radius *a*, having:

1 $$q_{i} = \frac{fit(i) - fitworst}{{fitbest - fitworst}}\quad i = 1,2, \ldots ,N$$

where, *fitbest* and *fitworst* are the best and worst objective function values among all of the particles, respectively; *fit*(*i*) represents the fitness of the agent *i*; and *N* is the total number of CPs.

The separation distance *r*_*ij*_ between any two CPs is defined as follows:

2 $$r_{ij} = \frac{{\left\| {X_{i} - X_{j} } \right\|}}{{\left\| {{{\left( {X_{i} + X_{j} } \right)} \mathord{\left/ {\vphantom {{\left( {X_{i} + X_{j} } \right)} 2}} \right. \kern-0pt} 2} - X_{best} } \right\| + \varepsilon }}$$

in which, *X*_*best*_ is the position of the best current CP, *X*_*i*_ is the position of the ith CP, and *ε* is a small positive number. Randomly positioned CPs are assumed to have zero velocities at their initial positions:

3 $$x_{ij}^{(0)} = x_{i,\min } + rand_{ij} .\left( {x_{i,\max } - x_{i,\min } } \right)\;,\quad i = 1,2,...,N$$

where, $x_{ij}^{(0)}$ determines the initial value of the *i*th variable for the *j*th CP; $x_{i,\min }$ and $x_{i,\max }$ are the minimum and maximum allowable values for the *i*th variable, respectively.

According to the following function, each CP is more likely to move towards the others:

4 $$P_{ij} = \left\{ \begin{gathered} 1\quad \quad \frac{fit(i) - fitbest}{{fit(j) - fit(i)}}> rand\; \vee \;fit(j)> fit(i) \hfill \\ 0\quad \quad otherwise \hfill \\ \end{gathered} \right.$$

In a spherical environment, CPs possess the ability to exert electric forces on one another. The magnitude of these forces varies based on the separation distance between CPs. For CPs situated within the sphere, the force’s magnitude relies directly on the separation distance. However, for CPs located outside the sphere, the force’s magnitude is inversely proportional to the square of the separation distance. The resultant force vector for each CP is calculated as follows:

5 $$F_{j} = q_{i} \sum\limits_{i,i \ne j} {\left( {\frac{{q_{i} }}{{a^{3} }}r_{ij} .i_{1} + \frac{{q_{i} }}{{r_{ij}^{2} }}.i_{2} } \right)P_{ij} \left( {X_{i} - X_{j} } \right)} \quad \left\langle \begin{gathered} j = 1,2, \ldots ,N \hfill \\ i_{1} = 1,i_{2} = 0 \Leftrightarrow r_{ij} < a \hfill \\ i_{1} = 0,i_{2} = 1 \Leftrightarrow r_{ij} \ge a \hfill \\ \end{gathered} \right.$$

where, *F*_*j*_ is the resultant force acting on the *j*th CP. The new location of the CPs is determined by the resultant forces and laws of motion. As a result of the resultant forces and its previous velocity, each CP moves toward its new position as follows^23^,:

6 $${X}_{j,new}=Fix\left(ran{d}_{j1}.{k}_{a}.\frac{{F}_{j}}{{m}_{j}}.\Delta {t}^{2}+ran{d}_{j2}.{k}_{v}.{V}_{j,old}.\Delta t+{X}_{j,old}\right)$$

7 $${V}_{j.new}=\frac{{X}_{j,new}-{X}_{j,old}}{\Delta t}$$

The acceleration and velocity coefficients are *k*_*a*_ and *k*_*v*_, respectively; and rand_*j*1_ and rand_*j*2_ are two random numbers evenly distributed in the range (0,1). The harmony search-based handling approach will correct the position of each CP if it deviates from the predefined bounds^5^. Using this method, any variable of each solution (*x*_*i,j*_) that violates its boundary can be regenerated from charged memory as follows:

8 $${x}_{i,j}=\left\{\begin{array}{c}w.p. CMCR\hspace{1em}\hspace{1em}\hspace{1em}\hspace{1em}\Rightarrow {\text{S}}{\text{e}}{\text{l}}{\text{e}}{\text{c}}{\text{t}}\hspace{0.33em}a\hspace{0.33em}{\text{n}}{\text{e}}{\text{w}}\hspace{0.33em}{\text{v}}{\text{a}}{\text{l}}{\text{u}}{\text{e}}\hspace{0.33em}{\text{f}}{\text{o}}{\text{r}}\hspace{0.33em}a\hspace{0.33em}{\text{v}}{\text{a}}{\text{r}}{\text{i}}{\text{a}}{\text{b}}{\text{l}}{\text{e}}\hspace{0.33em}{\text{f}}{\text{r}}{\text{o}}{\text{m}}\hspace{0.33em}{\text{C}}{\text{M}}\\ \hspace{1em}\hspace{1em}\hspace{1em}\hspace{1em}\hspace{1em}\hspace{1em}\hspace{0.33em}\hspace{1em}\hspace{1em}\hspace{0.33em}\Rightarrow {\text{w}}\text{.}{\text{p}}\text{.} \, ({1}-{\text{P}}{\text{A}}{\text{R}})\hspace{0.33em}{\text{d}}{\text{o}}\hspace{0.33em}{\text{n}}{\text{o}}{\text{t}}{\text{h}}{\text{i}}{\text{n}}{\text{g}}\hspace{0.33em}\\ \hspace{1em}\hspace{1em}\hspace{1em}\hspace{1em}\hspace{1em}\hspace{1em}\hspace{1em}\hspace{1em}\hspace{0.33em}\hspace{0.33em}\Rightarrow {\text{w}}\text{.}{\text{p}}\text{.} \, {\text{P}}{\text{A}}{\text{R}}\hspace{0.33em}{\text{c}}{\text{h}}{\text{o}}{\text{o}}{\text{s}}{\text{e}}\hspace{0.33em}a\hspace{0.33em}{\text{n}}{\text{e}}{\text{i}}{\text{g}}{\text{h}}{\text{b}}{\text{o}}{\text{r}}{\text{i}}{\text{n}}{\text{g}}\hspace{0.33em}{\text{v}}{\text{a}}{\text{l}}{\text{u}}{\text{e}}\\ w.p. \left(1-CMCR\right)\hspace{1em}\hspace{1em}\Rightarrow {\text{S}}{\text{e}}{\text{l}}{\text{e}}{\text{c}}{\text{t}}\hspace{0.33em}a\hspace{0.33em}{\text{n}}{\text{e}}{\text{w}}\hspace{0.33em}{\text{v}}{\text{a}}{\text{l}}{\text{u}}{\text{e}}\hspace{0.33em}{\text{r}}{\text{a}}{\text{n}}{\text{d}}{\text{o}}{\text{m}}{\text{l}}{\text{y}}\end{array}\right.$$

With the probability of *CMCR*, historical values stored in the charged memory (CM) are utilized to select a value in a new vector; while with a probability of (1-*CMCR*), a random value is selected from a possible range of values. With a probability of *PAR*, a random value is selected from the neighbourhood of the best CP, while with a probability of (1-*PAR*), a random value is selected from a predefined range of the variable. In summary, the pseudo-code for CSS appears as follows:

1. Initialize parameters: random positions of CPs, uniform volume charge density, and velocities.
2. Compare CPs based on fitness function values and sort them in ascending order.
3. Store CMS numbers of top CPs in the memory (CM) along with corresponding objective function values.
4. Calculate the probability of each CP moving towards others (Eq. 4).
5. Calculate the vector to attract each CP (Eq. 5).
6. Update positions and velocities of CPs (Eqs. 6,7).

If CP leaves search space, correct its position (Eq. 8).

7. Evaluate objective function values for new CPs and sort them.
8. Update CM: add the best CPs, remove the worst CPs.
9. Repeat steps 4–8 until termination criterion is met.

## Rank-based charged system search algorithm

At the outset of CSS, distant CPs experience an inversely proportional force due to the small value of parameter *k*_*a*_, fostering exploration with more searches in early iterations. As iterations progress, exploitation increases with a higher number of iterations, causing CPs to converge closer. Consequently, the resultant force shifts from inverse square to proportional to distance, necessitating a gradual increase in *k*_*a*_. To prevent excessive force on closer CPs, a selection process minimizes CP involvement, culminating in the CSSRank algorithm outlined below.

Initially, a parameter known as *Sel*_*(it)*_ is introduced to regulate the percentage of CPs involved in each iteration. This parameter follows a linear decreasing function, influencing the number of CPs exerting force on others. Higher values of *Sel*_*(it)*_ in early iterations facilitate exploration to discover the optimal solution. Conversely, lower values in later iterations enhance exploitation by focusing on the local space surrounding the current global best solution. The calculation of *Sel*_*(it)*_ values is outlined below:

9 $$\begin{gathered} Sel_{(it)} = Sel_{(i)} - \left[ {Sel_{(i)} - Sel_{(f)} } \right]\frac{it}{{MaxIt}} \hfill \\ or \hfill \\ Sel_{(it)} = Sel_{(f)} + \left[ {Sel_{(i)} - Sel_{(f)} } \right]\frac{MaxIt - it}{{MaxIt}} \hfill \\ \end{gathered}$$

where *Sel*_*(i)*_ is the initial value of the selected percentage; *Sel*_*(f)*_ is the final value of the selected percentage; *MaxIt* is the maximum number of iterations; and *it* is the current iteration number. The values of *Sel*_*(it)*_ get updated and decrease linearly from *Sel*_*(i)*_ to *Sel*_*(f)*_ by increasing the during the optimization process.

Setting *Sel*_*(i)*_ to 1 and *Sel*_*(f)*_ to 0.5 means that 100% of the CPs contribute to the total force on a CP in the initial iteration, and as the iterations progress, this contribution decreases gradually, with only 50% of the CPs participating in the force calculation in the final iteration. Thus, employing a linear decreasing function from 1 to 0.5 enhances the algorithm’s exploitation capability in the later stages of optimization.

After determining the number of selected CPs, the CSSRank algorithm employs a selection scheme to determine which CPs are selected. This scheme can utilize one of three methods: tournament selection, roulette wheel selection, or random selection.

- Tournament selection

In the tournament selection method, the parameter “Tour” defines the size of each tournament, typically ranging from 2 to *N*. Initially, several tournaments of CPs, each consisting of a specified number of individuals, are randomly selected. Subsequently, the individual with the highest fitness within each tournament is chosen.

- ***Roulette wheel selection***

In the roulette wheel selection method, CPs are represented as contiguous segments along a line, with each CP’s segment proportional in size to its fitness. A random number is then generated, and the CP whose segment encompasses the random number is chosen. This approach offers a probabilistic selection mechanism where every CP has a chance of being selected, mirroring natural selection processes found in nature.

- Random selection

In the random selection method, a CP is chosen randomly from all available CPs, without considering its fitness value. This approach introduces no task-driven selection pressure, as CPs are selected purely by chance.

The process of each selection method is repeated until the desired number of CPs is obtained ($Sel_{(it)} \times N$). It’s important to note that to prevent rapid convergence and avoid getting stuck in local optima, each CP can only be selected once in the current iteration. Algorithm 1 provides the detailed procedure for the rank-based reduction selection strategy used in *CSSRank* to dynamically control the number of charged particles (CPs) contributing to force calculations.

**Figure:**
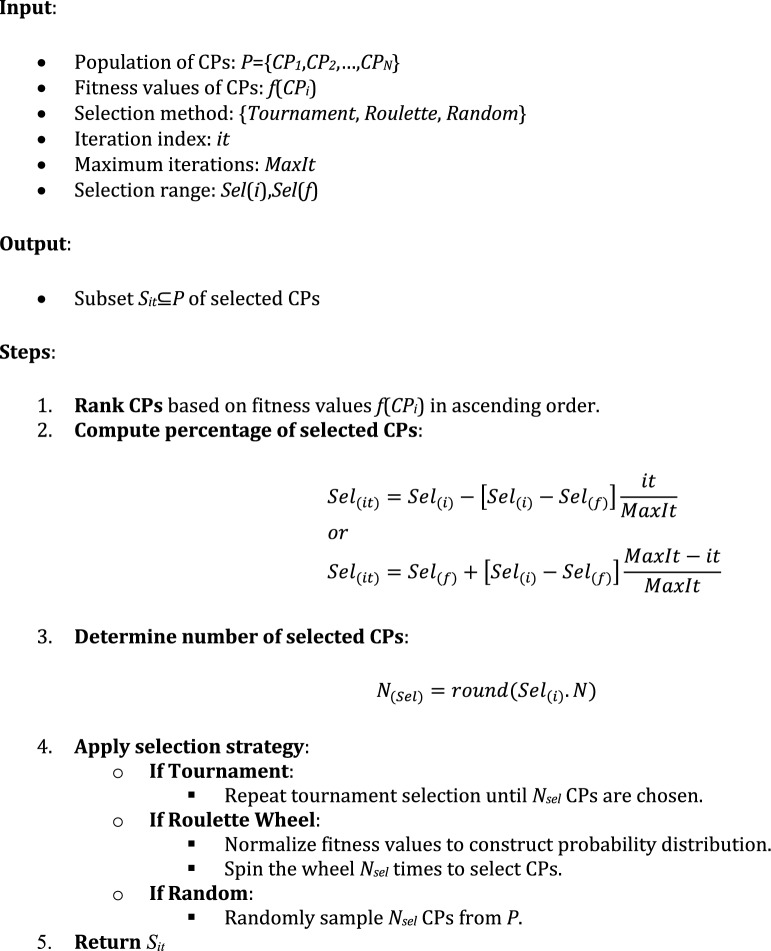
Algorithm 1: Rank-based reduction selection strategy

Additionally, we introduce a ranking-based mutation operator for CSSRank, where a proportion of the CPs are selected for mutation based on their rankings in the current iteration. This approach is outlined in detail below.

This step introduces a change probability parameter (*cpp*) within the range of (0, 1), determining whether a component of each CP needs to be altered. For each selected charged particle, a random number (*rnd*) is generated from a uniform distribution within the range of (0, 1). If *rnd* is less than *cpp (rnd*_*i*_ < *cpp)*, one dimension of the *i*th CP is randomly selected. To preserve the structure of the CPs, only one dimension of each CP is modified. Additionally, the parameters of the Harmony Search (HS)-based position correction method are disregarded for the final adjustment. Thus, Eq. 8 is revised as follows:

10 $$x_{i,j} = \left\{ \begin{gathered} \quad \quad \quad \quad \quad \quad \Rightarrow \left( 1 \right){\text{:Select}}\;{\text{a}}\;{\text{new}}\;{\text{value}}\;{\text{for}}\;{\text{a}}\;{\text{variable}}\;{\text{from}}\;{\text{CM}} \hfill \\ w.p.RS\quad \quad \;\;\; \Rightarrow \left( 2 \right){\text{:Choose a neighboring value}}\; \hfill \\ \quad \quad \quad \quad \quad \quad \Rightarrow \left( 3 \right){\text{:Select}}\;{\text{a}}\;{\text{new}}\;{\text{value}}\;{\text{randomly}} \hfill \\ \end{gathered} \right.$$

where *RS* is the rate of success. In the initial iteration, the probability of selecting a value from any of the three methods for the new vector is uniform and set to 0.33%. However, in subsequent iterations, the rate of success determines the probability. If the choices made result in solution improvements, the probability increases; otherwise, it decreases. Algorithm 2 outlines the ranking-based mutation strategy employed in CSSRank, where a subset of CPs is probabilistically modified based on their rank and an adaptive change probability.

**Figure:**
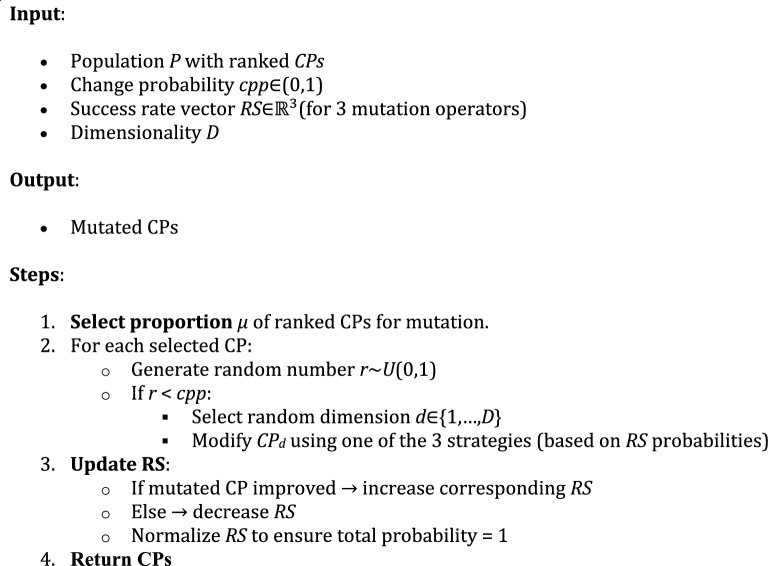
Algorithm 2: Ranking-based mutation operator.

The steps of the CSSRank algorithm are as follows:

## Step 1: Initialization

*Initialize Parameters:* Set up the CSSRank algorithm parameters.

*Initialize Charged Particles*: Generate random CPs and their corresponding velocities.

*Rank CPs:* Compare the fitness function values for the CPs and sort them in ascending order.

*Create Charge Memory (CM):* Store the CMS numbers of the first CPs in the CM.

## Step 2: Main loop

*Select CPs for Attraction Force:* Choose a percentage of CPs using the selection methods for calculating the attraction force.

*Determine Attracting Force:* Calculate the attracting force vector for each CP based on the probability of moving towards other CPs.

*Generate New Solutions:* Move each CP to a new position and update their velocities.

*Correct CP Positions:* If any CP exits the allowable search space, adjust its position.

*Rank CPs:* Evaluate and compare the objective function values for the new CPs, sorting them in ascending order.

*Update Charge Memory:* Include better CP vectors in the CM and remove worse ones.

*Update Best and Worst CPs:* Assign the first and last CPs to the global best and worst CPs, respectively, up to the current iteration.

*Update Selecting Percentage*: Adjust the selecting percentage parameter.

## Step 3: Mutation

*Apply Mutation Operator:* Apply the mutation operator to a selected number of CPs.

## Step 4: Truncation

*Merge and Truncate CPs:* Merge CPs obtained from mutation, sort and select the best CPs.

## Step 5: Termination criterion control

*Repeat Iterations:* Repeat the above steps until a termination criterion is met.

**Fig. 1:**
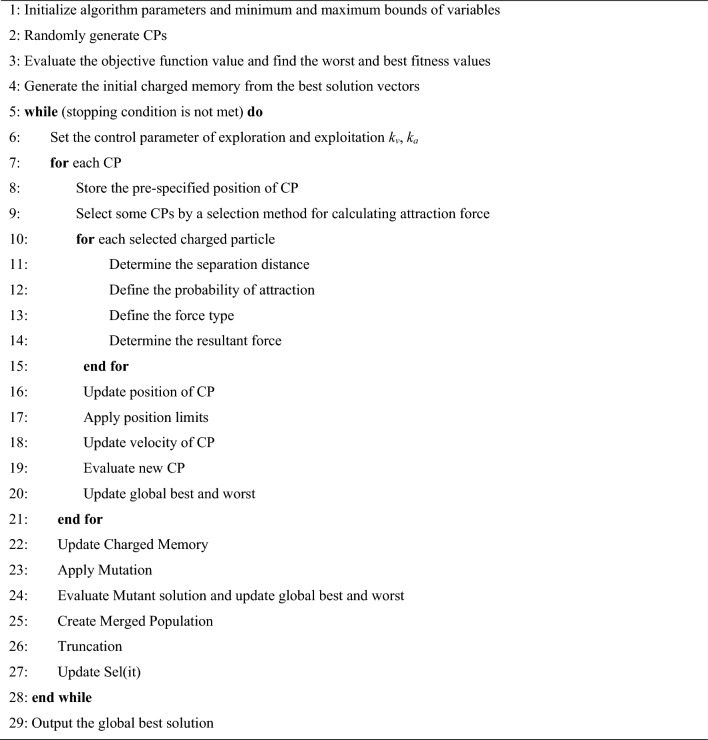
Pseudo-code of the CSSRank algorithm.

**Fig. 2.**
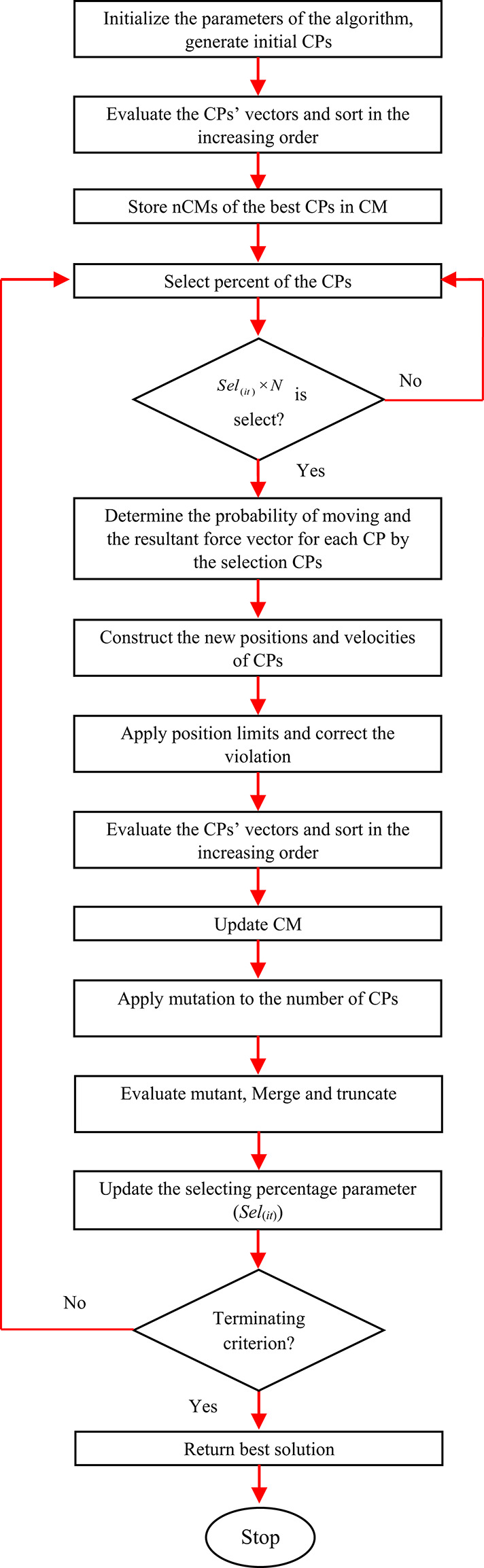
Flowchart of the CSSRank algorithm.

Based on the outlined steps, the CSSRank algorithm is encapsulated in a pseudo-code provided in Fig. 1. Additionally, the flowchart representation of the CSSRank algorithm can be observed in Fig. 2.

The proposed improvements to the CSS algorithm enhance its performance by balancing exploration and exploitation. Reducing the number of CPs for force estimation increases exploitation in the final steps, while ranking-based mutation boosts exploration. Simplifying the CP position correction process and eliminating fixed parameters reduces complexity but slightly slows convergence due to increased calculations. Overall, these changes significantly improve the algorithm’s optimization efficiency.

The incorporation of the *Sel*(*it*) parameter to reduce the number of CPs involved in estimating total force enhances the algorithm’s exploitation capability in later optimization stages. Additionally, employing ranking-based mutation techniques enhances exploration. These adjustments strike a balance between exploration and exploitation, leading to significant performance improvements. Furthermore, simplifying the algorithm’s structure by altering CPs position correction and eliminating available parameters reduces complexity. Overall, these modifications significantly enhance the CSS algorithm’s convergence performance in optimization tasks.

## Mathematical numerical experiments

In this section, we present a set of mathematical benchmark functions and compare the performance of the proposed method with several well-established optimization algorithms. Sub-section "Mathematical benchmark functions" evaluates the performance of the CSSRank algorithm on 18 standard mathematical benchmark functions. The algorithm’s ability to reach global optima is assessed by analyzing the number of function evaluations (NFE) required. Additionally, this section introduces a methodology for visualizing the exploration–exploitation balance using normalized performance metrics, providing empirical insights into the optimization dynamics of CSSRank.

Sub-section "CEC2014 problems" extends the evaluation to a set of benchmark functions from the CEC 2014 Special Session on Real-Parameter Optimization, organized by the IEEE Congress on Evolutionary Computation^32^. This section presents comparative analyses between CSSRank and several established algorithms, illustrating relative performance in terms of accuracy and convergence behavior.

Sub-section "CEC 2024 problems" focuses on the most recent benchmark suite, CEC 2024^33^, and compares CSSRank against a range of state-of-the-art optimization algorithms. The results demonstrate CSSRank competitiveness with the best-performing methods in the literature, highlighting its robustness and modern relevance.

### Mathematical benchmark functions

In this section, we conducted a thorough examination of the performance of CSSRank algorithm by optimizing well-studied mathematical benchmarks selected from the literature^23^. We specifically focused on analyzing the impact of key parameters such as population size, problem dimensionality, mutation parameter, selection methods and mutation strategy on the algorithm’s efficacy. For clarity and ease of understanding, we chose two benchmark functions from the literature to assess the convergence rate of the developed algorithm. The characteristics of these selected functions are detailed in Table 2, providing valuable insights into their complexities and properties. To evaluate the performance of CSSRank across different parameter configurations, we conducted extensive experiments simulating various parameter settings. The results obtained from these experiments were then compared with those of the standard CSS algorithm, serving as a benchmark for performance assessment. Two termination criteria were employed to conclude the algorithms’ execution: 1) reaching a predefined maximum number of iterations (a constant value), and 2) achieving a minimum error threshold. In our study, we set the maximum number of iterations to 500, and any error value less than 10^−18^ was recorded as 0 to facilitate analysis and interpretation. To ensure the robustness and reliability of the findings, we conducted 50 independent runs for each algorithm, each initiated with distinct initial conditions. This approach allowed us to comprehensively assess the algorithm’s performance under various scenarios and ascertain its robustness across different settings.

**Table 2:** Specifications of the benchmark problems. D: dimension, C: characteristic, U: unimodal, M: multimodal.

Name	Function	C	D	Range	Global minimum
Griewank	$f\left( X \right) = 1 + \frac{1}{200}\sum\limits_{i = 1}^{n} {x_{i}^{2} } - \prod\limits_{i = 1}^{n} {\cos \left( {\frac{{x_{i} }}{\sqrt i }} \right)}$	M	10	X∈[−600,600]^n^	0.0
Ackley	$f\left( X \right) = - 20\exp \left( { - 0.2\sqrt {\frac{1}{n}\sum\limits_{i = 1}^{n} {x_{i}^{2} } } } \right) - \exp \left( {\sqrt {\frac{1}{n}\sum\limits_{i = 1}^{n} {\cos \left( {2\pi x_{i} } \right)} } } \right) + 20 + e$	M	10	X∈[−32.8,32.8]^n^	0.0

**Fig. 3:**
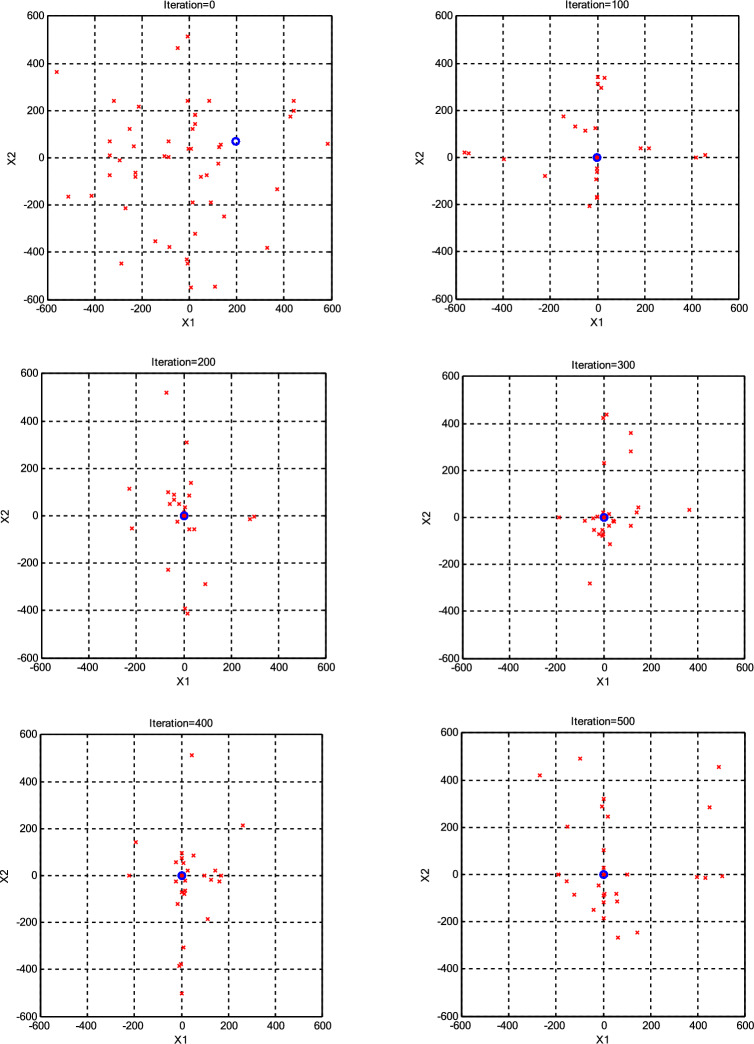
The positions of the current CPs and the best CP in CSS for the Griewank function.

**Fig. 4:**
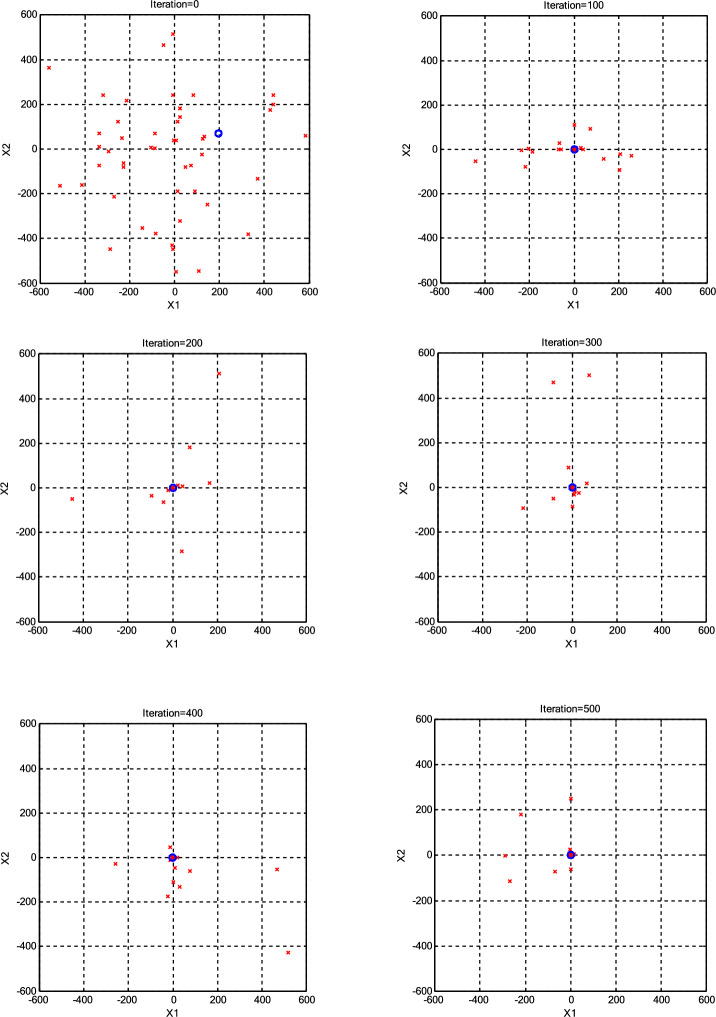
The positions of the current CPs and the best CP in CSSRank for the Griewank function.

**Fig. 5:**
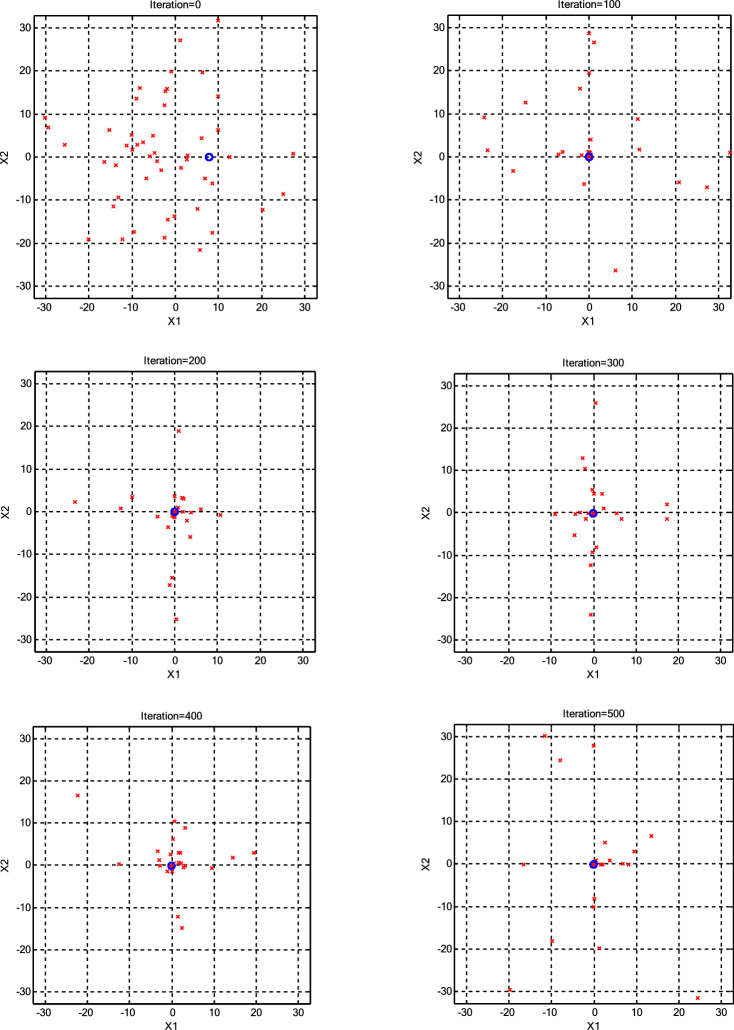
The positions of the current CPs and the best CP in CSS for the Ackley function.

**Fig. 6:**
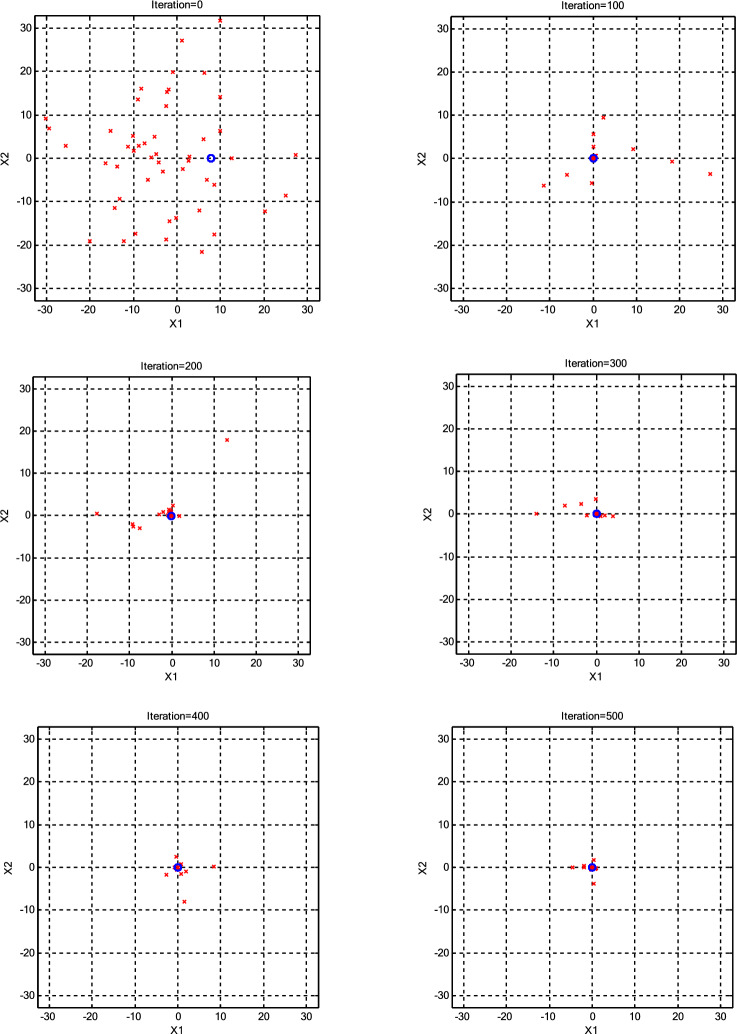
The positions of the current CPs and the best CP in CSSRank for the Ackley function.

The impact of population size (*N*) on the optimization process was assessed, as presented in Table 3. The minimum error achieved by each algorithm for every *N* is highlighted in bold for clarity. Interestingly, it was observed that a population size of 30 CPs consistently outperformed both larger and smaller populations across various scenarios. Consequently, we maintained a constant population size (*N* =30) for further parameter variations. Additionally, it was noted that the roulette wheel selection method exhibited superior performance compared to the other two selection methods for the Griewank function. Conversely, for the Ackley function, optimal results were obtained with different selection methods depending on the specific population size, with tournament selection proving optimal for *N* =10 and *N* =20, roulette wheel for *N* =30, a combination of roulette wheel and tournament for *N* =40, and random selection for *N* =50. To provide a more transparent illustration of algorithm performance under high CP counts (N=50), Figs. 3,4,5,6 visualize the positions of the current CPs and the best CP in both CSS and CSSRank for the Griewank and Ackley functions. Notably, in the final iterations, CSSRank consistently outperforms the standard CSS algorithm, as evidenced by improved convergence and proximity to optimal solutions.

**Table 3:** Variation of mean and standard deviation (±SD) of error with different *N* (dimension=10).

Function	*N*	Standard CSS	CSSRank		
			Roulette	Tornament	Random
Griewank	10	1.22E+0 (7.89E-1)	**4.74E-1 (3.71E-1)**	4.86E-1 (4.15E-1)	5.37E-1 (4.83E-1)
	20	3.85E-2 (8.12E-2)	**5.90E-3 (1.32E-2)**	9.90E-3 (2.94E-2)	2.06E-2 (4.18E-2)
	30	2.88E-3 (4.21E-2)	**1.47E-4 (4.66E-4)**	1.30E-3 (3.70E-3)	5.71E-4 (1.80E-3)
	40	1.46E-2 (4.60E-2)	**3.88E-4 (1.22E-4)**	2.10E-3 (5.70E-3)	5.79E-3 (6.01E-3)
	50	2.87E-2 (9.06E-2)	**6.55E-4 (2.10E-3)**	3.70E-3 (1.18E-2)	1.62E-2 (4.58E-2)
Ackley	10	9.88E+0 (2.12E+0)	2.73E+0 (9.19E-1)	**1.39E+0 (1.24E+0)**	2.69E+0 (1.71E+0)
	20	9.74E-1 (1.14E+0)	7.70E-1 (9.33E-1)	**1.06E-1 (2.73E-1)**	1.50E-1 (3.59E-1)
	30	3.01E-3 (1.09E-2)	**5.86E-15 (1.83E-15)**	6.22E-15 (1.87E-15)	6.22E-15 (1.87E-15)
	40	1.65E-1 (5.21E-1)	**5.86E-15 (1.83E-15)**	6.93E-15 (3.76–15.76)	**5.86E-15 (1.83E-15)**
	50	3.34E-1 (7.46E-1)	6.93E-15 (1.72E-15)	9.06E-15 (2.40E-15)	**6.22E-15 (1.87E-15)**

Another influential parameter affecting the performance of the proposed algorithm is the problem dimensionality. The benchmarks were tested with dimensions of 2, 10, 20, and 30 by CSSRank with different selection methods. Table 4 presents the average optimal value and standard deviation of optimal values for *N*=30 for each function. The obtained results indicate that for a constant population size, as the dimension of the problem increases, the performance of the algorithms decreases.

**Table 4:** Variation of mean and standard deviation (±SD) of error with variation of dimension (*N*=30).

Function	Dimension	Standard CSS	CSSRank		
			Roulette	Tornament	Random
Griewank	2	4.31E-5 (±9.08E-5)	0 (0)	0 (0)	0 (0)
	10	2.88E-3 (±4.21E-2)	1.47E-4 (±4.66E-4)	1.30E-3 (±3.70E-3)	5.71E-4 (±1.80E-3)
	20	1.06E-1 (±1.31E-1)	9.00E-3 (±2.12E-2)	1.31E-1 (±2.30E-1)	3.40E-3 (±1.07E-2)
	30	2.63E+1 (±5.51E+1)	7.82E-1 (±5.89E-1)	1.39E+0 (±9.35E-1)	6.24E-1 (±5.24E-1)
Ackley	2	7.33E-6 (±2.11E-5)	8.18E-16 (0)	8.18E-16 (0)	1.24E-15 (±1.12E-15)
	10	3.01E-3 (±1.09E-2)	5.86E-15 (±1.83E-15)	6.22E-15 (±1.87E-15)	6.22E-15 (±1.87E-15)
	20	2.81E+0 (±3.39E+0)	2.34E-1 (±2.85E-1)	9.10E-1 (±7.08E-1)	3.77E-1 (±5.33E-1)
	30	9.39E+1 (±2.01E+2)	1.97E+0 (±8.88E-1)	1.79E+0 (±7.24E-1)	1.78E+0 (±1.08E+0)

Furthermore, the mutation operator helps the algorithm balance between global and local search and avoid trapping in local minima. This balance can be achieved by the mutation percentage parameter (*pm*). Table 5 shows the mean and standard deviation of error with the variation of mutation percentage on the two benchmarks for *N*=30 and *D*=10. The best results were obtained when *pm*=0.1.

**Table 5:** Variation of mean and standard deviation (±SD) of error with variation of *pm* (dimension=10, *N*=30).

Function	Standard CSS	Mutation percentage	CSSRank		
			Roulette	Tornament	Random
Griewank	2.88E-3 (±4.21E-2)	0	2.30E-3 (±7.40E-3)	2.61E-3 (±1.13E-2)	3.11E-3 (±4.54E-2)
		0.01	1.86E-4 (±5.25E-4)	2.39E-3 (±1.49E-2)	2.92E-3 (±2.50E-2)
		0.05	1.52E-4 (±4.58E-4)	1.77E-3 (±6.01E-3)	2.03E-3 (±1.39E-2)
		0.1	**1.47E-4 (±4.66E-4)**	**1.30E-3 (±3.70E-3)**	**5.71E-4 (±1.80E-3)**
		0.15	3.31E-4 (±1.00E-3)	4.30E-3 (±1.29E-2)	3.00E-3 (±8.00E-3)
Ackley	3.01E-3 (±1.09E-2)	0	1.15E-3 (±3.65E-3)	2.31E-3 (±4.87E-3)	1.65E-3 (±5.21E-3)
		0.01	2.61E-13 (±7.57E-13)	6.01E-12 (±4.21E-12)	8.91E-13 (±8.42E-13)
		0.05	4.44E-14 (±2.21E-13)	7.21E-13 (±5.49E-13)	1.91E-13 (±1.10E-13)
		0.1	**5.86E-15 (±1.83E-15)**	**6.22E-15 (±1.87E-15)**	**6.22E-15 (±1.87E-15)**
		0.15	6.22E-15 (±1.87E-15)	8.32E-15 (±2.86E-15)	7.59E-15 (±3.40E-15)

As previously mentioned, the type of selection method is another crucial factor of meta-heuristic algorithms. For this assessment, the number of variables and *N* were set to 10 and 30, respectively. Statistical analyses of the fitness values obtained through simulation runs for benchmark functions using different selection methods for the CSSRank algorithm were performed. The results in Table 6 reveal the superiority of the gradually reduced CPs strategy over three selection methods for CSSRank compared to the standard CSS. Based on the results, the relative superiority of the roulette wheel selection method to the other methods is proven, even though all selection methods were capable of reaching the best minimum value. Therefore, the roulette wheel selection method was considered for solving subsequent problems.

**Table 6:** Statistical results of the selection methods for benchmark functions for the CSSRank algorithm.

Function	Algorithm		Best	Mean	Median	Worst	Std.Dev.
Griewank	Standard CSS		8.86E-06	2.88E-3	1.15E-3	1.55E-2	4.21E-2
	CSSRank	Roulette wheel	0	1.47E-4	0	1.50E-3	4.66E-4
		Tornament	0	1.30E-3	0	1.16E-2	3.70E-3
		random	0	5.71E-4	0	5.70E-3	1.80E-3
Ackley	Standard CSS		8.10E-5	3.01E-3	4.90E-4	4.91E-2	1.09E-2
	CSSRank	Roulette wheel	4.44E-15	5.86E-15	4.44E-15	7.99E-15	1.83E-15
		Tornament	4.44E-15	6.22E-15	6.22E-15	7.99E-15	1.87E-15
		Random	4.44E-15	6.22E-15	6.22E-15	7.99E-15	1.87E-15

The rank-based mutation strategy integrated into CSSRank is specifically designed to balance exploration and exploitation by selectively applying controlled mutations to elite particles, with dynamic adjustment of mutation probability over time. Unlike traditional strategies such as Uniform Mutation (random value replacement), Gaussian Mutation (zero-mean noise), and Non-uniform Mutation (gradually decreasing step size), the proposed method applies changes to only one dimension per selected particle and linearly reduces the mutation probability across iterations. This mechanism preserves population diversity in early stages while refining promising regions later in the search. To evaluate its effectiveness, we compared CSSRank variants incorporating No Mutation, Uniform Mutation, and Gaussian Mutation across four benchmark functions: Sphere, Rastrigin, Griewank, and Ackley (Table 7). Key findings include:

- On Rastrigin, the proposed method achieved the best Best-case result (1.42E-14) and the lowest standard deviation, indicating strong consistency and global search capacity.
- On Sphere, it demonstrated faster convergence (Mean = 3.12E-11, Std. Dev. = 2.08E-10), reflecting superior exploitation.
- On Griewank, it maintained the tightest result distribution (Mean = 5.95E-06).
- On Ackley, it delivered the most stable and accurate outcomes (Mean = 1.31E-03, Std. Dev. = 6.29E-03).

**Table 7:** Statistical results of the mutation strategies for benchmark functions for the CSSRank algorithm.

Function	Mutation strategy	Best	Mean	Median	Worst	Std.Dev.
Sphere	No mutation	**9.34E-28**	6.71E-10	1.47E-16	1.81E-08	2.90E-09
	Uniform mutation	5.49E-27	1.22E-10	1.29E-18	6.09E-09	8.61E-10
	Gaussian mutation	1.42E-27	**8.41E-12**	**1.12E-18**	4.12E-10	**5.83E-11**
	Rank-based mutation	2.11E-27	3.12E-11	2.44E-17	1.47E-09	2.08E-10
Rastrigin	No mutation	5.97E+00	1.60E+01	1.39E+01	3.28E+01	7.29E+00
	Uniform mutation	6.39E-07	4.18E-01	1.45E-01	2.60E+00	5.97E-01
	Gaussian mutation	3.98E+00	1.47E+01	1.34E+01	3.23E+01	6.11E+00
	Rank-based mutation	**1.42E-14**	**2.93E-01**	**8.05E-02**	2.60E+00	**4.76E-01**
Griewank	No mutation	0.00E+00	9.39E-02	2.71E-02	9.28E-01	2.22E-01
	Uniform mutation	0.00E+00	2.68E-04	6.94E-14	1.23E-02	1.74E-03
	Gaussian mutation	0.00E+00	3.38E-02	1.97E-02	2.24E-01	4.62E-02
	Rank-based mutation	**0.00E+00**	**5.95E-06**	**1.34E-14**	1.82E-04	**2.87E-05**
Ackley	No mutation	2.84E-12	1.15E+00	1.16E+00	3.03E+00	8.37E-01
	Uniform mutation	1.71E-13	6.65E-03	1.13E-08	1.11E-01	2.29E-02
	Gaussian mutation	4.45E-12	9.01E-01	5.78E-01	3.57E+00	1.01E+00
	Rank-based mutation	**2.00E-13**	**1.31E-03**	**1.30E-09**	4.13E-02	**6.29E-03**

**Fig. 7:**
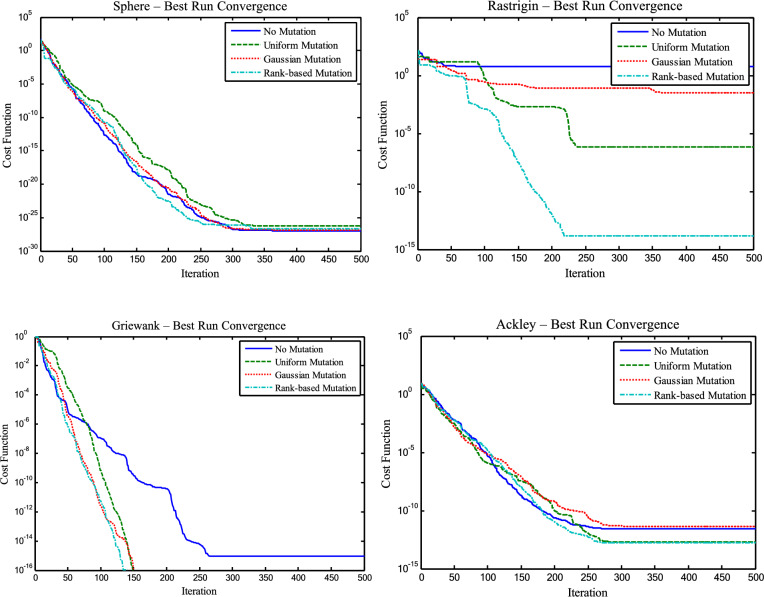
Convergence curves of best value for the mutation strategies for benchmark functions for the CSSRank algorithm.

In addition, Figure 7 illustrates that while all methods eventually converge on unimodal functions, the rank-based mutation converges faster, and in multimodal functions, it exhibits sharper early descent and escapes local optima more effectively.

In this subsetion, we investigate Exploration-Exploitation (EE) balance for the developed method as well. To fulfill this aim, we use normalized metrics of population spread for exploration and proximity to the best solution for exploitation, computed over 500 iterations and 50 independent runs. These metrics are defined as:

Exploration

11 $$E_{\exp } \left( t \right) = \frac{1}{D}\sum\limits_{j = 1}^{D} {{\text{std}} \left( {X_{:,j}^{\left( t \right)} } \right)}$$

where ${X}_{:,j}^{\left(t\right)}$ is the vector of CP positions in dimension *j*, and $\mathit{std}\left(.\right)$ measures population spread, reflecting exploratory behavior.

Exploitation

12 $$E_{{{\text{expl}}}} \left( t \right) = \frac{1}{N}\sum\limits_{i = 1}^{N} {\left\| {x_{i}^{\left( t \right)} - x_{best} } \right\|}_{2}$$

where ${x}_{i}^{\left(t\right)}$ is the position particle *i* at iteration t, and ${x}_{best}$ is the globally best solution, indicating local improvement focus. Raw metrics are normalized to [0,1] for comparability:

13 $$E_{\exp }^{N,\left( r \right)} \left( t \right) = \frac{{E_{\exp }^{\left( r \right)} \left( t \right) - \min_{{\tau = 1,...,{\text{MaxIt}}}} E_{\exp }^{\left( r \right)} \left( \tau \right)}}{{\max_{{\tau = 1,...,{\text{MaxIt}}}} E_{\exp }^{\left( r \right)} \left( \tau \right) - \min_{{\tau = 1,...,{\text{MaxIt}}}} E_{\exp }^{\left( r \right)} \left( \tau \right) + \varepsilon }}$$

14 $$E_{{{\text{expl}}}}^{N,\left( r \right)} \left( t \right) = 1 - \frac{{E_{{{\text{expl}}}}^{\left( r \right)} \left( t \right) - \min_{{\tau = 1,...,{\text{MaxIt}}}} E_{{{\text{expl}}}}^{\left( r \right)} \left( \tau \right)}}{{\max_{{\tau = 1,...,{\text{MaxIt}}}} E_{{{\text{expl}}}}^{\left( r \right)} \left( \tau \right) - \min_{{\tau = 1,...,{\text{MaxIt}}}} E_{{{\text{expl}}}}^{\left( r \right)} \left( \tau \right) + \varepsilon }}$$

In these expressions, ${E}_{exp}^{\left(r\right)\left(t\right)}$ and ${E}_{\text{expl}}^{\left(r\right)}\left(t\right)$ denote the raw exploration and exploitation metrics computed during the *r*-th independent run at iteration t, respectively. The iteration index τ ranges from 1 up to *MaxIt*, which specifies the maximum number of iterations (500). The operations ${min}_{\tau }$ and ${max}_{\tau }$ extract the minimum and maximum values of each metric over all iterations within the same run. The small constant ε prevents division by zero during normalization. Finally, *r* indexes each of the *R* independent runs (*R*=50), and after normalizing within each run, the metrics are averaged across these *R* runs to produce the final exploration–exploitation curves:

15 $$\overline{E}_{\exp }^{N} \left( t \right) = \frac{1}{R}\sum\limits_{r = 1}^{R} {E_{\exp ,r}^{N} \left( t \right)} \;,\quad \overline{E}_{{{\text{expl}}}}^{N} \left( t \right) = \frac{1}{R}\sum\limits_{r = 1}^{R} {E_{{{\text{expl}},r}}^{N} \left( t \right)}$$

**Fig. 8:**
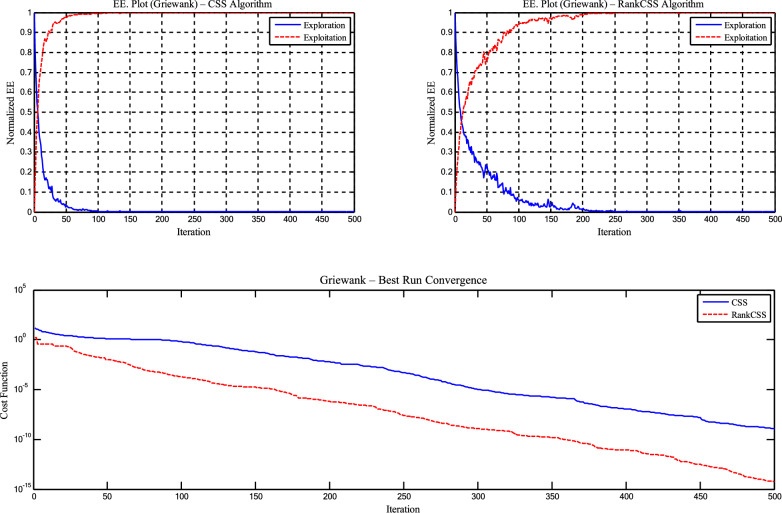
Exploration - exploitation and convergence curves of Griewank function for the CSS and CSSRank algorithms.

**Fig. 9:**
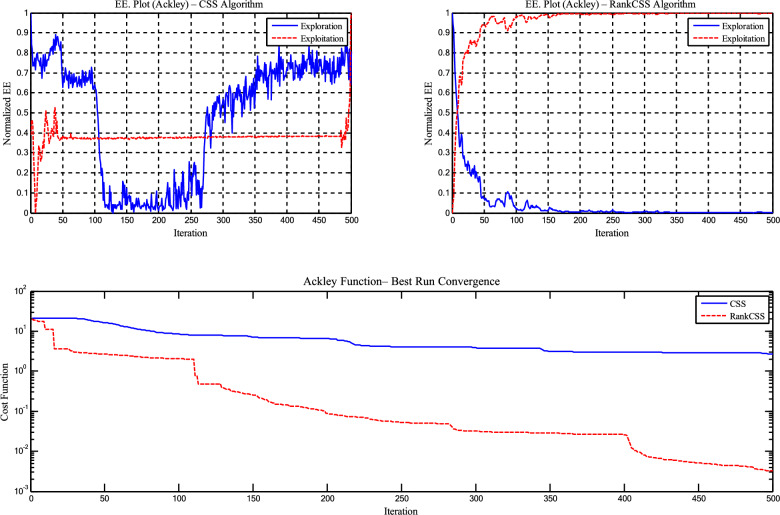
Exploration - exploitation and convergence curves of Ackley function for the CSS and CSSRank algorithms.

The *Sel*_*(it)*_ parameter and targeted mutation strategy in CSSRank contribute significantly to improving the exploration–exploitation (EE) balance, as demonstrated through experiments on the Griewank and Ackley functions with dimensionality *D*=50. CSSRank exhibits a gradual decline in exploration and a stable increase in exploitation over iterations, resulting in a Best value of 5.88E-15 on Griewank and 3.27E-03 on Ackley, compared to CSS’s 1.31E-09 and 1.17E+01, respectively. Furthermore, CSSRank achieved notably lower standard deviations, for example, 3.93E-02 vs. 8.90E-02 on the Griewank function, indicating more consistent convergence behavior. These results confirm the superior EE balancing capability of CSSRank, as detailed in Table 8 and illustrated in Figures 8 and 9.

**Table 8:** Statistical results of the EE for benchmark functions for the CSS and CSSRank algorithm.

Function	Algorithm	Best	Mean	Std. Dev. exploitation	Std. Dev. exploration
Griewank	Standard CSS	1.31E-09	2.70E-02	1.01E-01	8.90E-02
	CSSRank	5.88E-15	7.74E-13	3.30E-02	3.93E-02
Ackley	Standard CSS	2.70E+00	1.17E+01	4.62E-02	3.02E-01
	CSSRank	3.27E-03	5.25E-02	7.31E-03	8.75E-03

### CEC2014 problems

In this section, we conduct a comprehensive evaluation of CSSRank to investigate its computational power. We compare the performance of CSSRank with several other meta-heuristic algorithms using functions designed for the Special Session on Single Objective Real Parameter Numerical Optimization organized at the 2014 IEEE Congress on Evolutionary Computation (CEC 2014)^32^. The properties of the objective functions and their optimal values are summarized in Table 9.

**Table 9:** Summary of the CEC’14 Test functions.

NO.	Unimodal functions (3)	$F_{i}^{ * } = F_{i} \left( {x^{ * } } \right)$
1	Rotated high conditioned elliptic function	100
2	Rotated bent cigar function	200
3	Rotated discus function	300
NO.	Simple multimodal functions (13)	$F_{i}^{ * } = F_{i} \left( {x^{ * } } \right)$
4	Shifted and rotated Rosenbrock’s function	400
5	Shifted and rotated Ackley’s function	500
6	Shifted and rotated Weierstrass function	600
7	Shifted and rotated Griewank’s function	700
8	Shifted Rastrigin’s function	800
9	Shifted and rotated Rastrigin’s function	900
10	Shifted Schwefel’s function	1000
11	Shifted and rotated Schwefel’s function	1100
12	Shifted and Rotated Katsuura function	1200
13	Shifted and Rotated HappyCat function	1300
14	Shifted and Rotated HGBat function	1400
15	Shifted and rotated expanded Griewank’s plus Rosenbrock’s function	1500
16	Shifted and rotated expanded Scaffer’s F6 function	1600
NO.	Hybrid function 1 (6)	$F_{i}^{ * } = F_{i} \left( {x^{ * } } \right)$
17	Hybrid function 1 (N=3)	1700
18	Hybrid function 2 (N=3)	1800
19	Hybrid function 3 (N=4)	1900
20	Hybrid function 4 (N=4)	2000
21	Hybrid function 5 (N=5)	2100
22	Hybrid function 6 (N=5)	2200
NO.	Composition functions (8)	$F_{i}^{ * } = F_{i} \left( {x^{ * } } \right)$
23	Composition function 1 (N=5)	2300
24	Composition function 2 (N=3)	2400
25	Composition function 3 (N=3)	2500
26	Composition function 4 (N=5)	2600
27	Composition function 5 (N=5)	2700
28	Composition function 6 (N=5)	2800
29	Composition Function 7 (N=3)	2900
30	Composition function 8 (N=3)	3000
Search Range: [−100, 100]^D^		

The CEC 2014 test suite consists of 30 benchmark functions categorized into four classes: unimodal (Benchmarks 1–3), simple multimodal (Benchmarks 4–16), hybrid functions (Benchmarks 17–22), and composite functions (the rest). Following the parameters suggested by Liang et al.^32^, each function was solved using CSSRank in 30 independent runs. The mean and standard deviation of error were recorded. The evaluation was performed for dimensions 30 and 50, with a range of [−100, 100], and the maximum number of function evaluations was D × 10^3.

The termination criterion was defined as either reaching the maximum number of function evaluations or achieving an error less than 10^−8^, whichever was attained first. The error value is defined as the difference between the obtained objective function value and the desired function value ($F_{i}^{ * }$).

Table 10 presents the results of the six participating algorithms as obtained from and described in^34^. Additionally, Tables 10 and 11 display the results of the standard CSS and CSSRank algorithms using the roulette wheel selection method, where the best result in each row is marked in boldface. The CSSRank outperformed the other algorithms on the vast majority of benchmark functions over both dimensions. However, there were some instances where CSSRank performed slightly weaker than certain other algorithms. Specifically, CSSRank outperformed the others on 20 problems, while the EFO and CODE algorithms performed best on four problems each. The standard CSS and GSA algorithms found the best result for one problem each, while CLPSO, ABC, and GSO did not achieve the best outcome for any of the 30D functions (Table 10).

**Table 10:** The mean minimization value and standard deviation (SD) over 30 trials with D = 30.

Functions	Meas.	GSA	CLPSO	ABC	GSO	CoDE	EFO	CSS	CSSRank
$f_{1}$	Mean SD	3.14E+08 6.12E+07	1.20E+08 3.16E+07	3.08E+07 1.90E+07	2.20E+08 5.38E+07	7.81E+06 5.32E+06	**5.75E+05** **3.37E+05**	1.53E+07 1.02E+07	1.04E+07 7.08E+06
$f_{2}$	Mean SD	1.55E+10 3.15E+09	3.15E+09 7.96E+08	1.82E+04 1.24E+04	1.44E+10 2.10E+09	3.28E+07 1.32E+07	2.18E+02 5.60E+02	3.79E+02 3.55E+02	**1.14E+02** **6.50E+01**
$f_{3}$	Mean SD	8.64E+04 7.90E+03	4.04E+04 1.16E+04	2.13E+03 1.51E+03	9.96E+04 1.76E+04	**2.20E+02** **7.34E+01**	2.06E+03 2.11E+03	1.63E+4 6.49E+3	9.09E+03 5.30E+03
$f_{4}$	Mean SD	1.39E+03 2.38E+02	4.96E+02 7.33E+01	1.05E+02 2.63E+01	1.84E+03 2.51E+02	1.71E+02 1.34E+01	5.08E+01 4.18E+01	7.67E+01 4.60E+01	**3.79E+01** **2.73E+01**
$f_{5}$	Mean SD	**2.00E+01** **4.56E-04**	2.10E+01 7.07E-02	2.04E+01 4.71E-02	2.04E+01 8.21E-02	2.08E+01 4.55E-02	2.00E+01 2.99E-02	2.00E+01 6.29E-02	2.00E+01 5.63E-02
$f_{6}$	Mean SD	3.04E+01 2.47E+00	2.85E+01 1.47E+00	1.92E+01 1.61E+00	1.26E+02 6.15E+00	3.01E+01 1.40E+00	5.09E+00 1.59E+00	2.18E+00 2.69E+00	**1.46E+00** **2.91E+00**
$f_{7}$	Mean SD	1.63E+02 2.53E+01	2.92E+01 5.31E+00	3.12E-01 1.30E-01	9.62E+01 1.92E+01	1.30E+00 9.26E-02	1.02E-02 1.50E-02	1.62E-03 2.52E-03	**1.33E-03** **1.72E-03**
$f_{8}$	Mean SD	1.46E+02 9.73E+00	1.62E+02 1.27E+01	6.45E+00 1.59E+00	4.17E+02 3.38E+01	1.05E+02 7.50E+00	**9.29E-01** **9.03E-01**	5.48E+01 1.73E+01	4.27E+01 9.49E+00
$f_{9}$	Mean SD	1.64E+02 1.82E+01	2.51E+02 1.22E+01	1.25E+02 2.12E+01	8.09E+02 6.48E+01	2.00E+02 1.22E+01	1.55E+02 4.71E+01	6.54E+01 1.66E+01	**3.75E+01** **1.71E+01**
$f_{10}$	Mean SD	3.91E+03 6.06E+02	4.26E+03 2.91E+02	1.01E+02 7.57E+01	7.66E+03 1.01E+03	3.34E+03 2.25E+02	**3.08E+00** **1.53E+00**	1.38E+03 4.61E+02	1.11E+03 1.48E+02
$f_{11}$	Mean SD	4.38E+03 5.09E+02	6.30E+03 4.23E+02	2.90E+03 2.51E+02	1.76E+04 1.39E+03	6.17E+03 2.72E+02	7.25E+03 2.53E+02	**4.47E+02** **1.65E+02**	7.28E+02 3.73E+02
$f_{12}$	Mean SD	2.03E-02 1.14E-02	2.47E+00 3.52E-01	4.66E-01 8.11E-02	1.14E+00 2.12E-01	1.72E+00 2.22E-01	2.99E+00 3.85E-01	2.85E-02 1.71E-02	**1.89E-02** **1.26E-02**
$f_{13}$	Mean SD	3.73E+00 2.88E-01	6.79E-01 8.40E-02	3.82E-01 4.32E-02	5.54E-01 4.85E-02	5.86E-01 8.43E-02	3.01E-01 6.25E-02	2.82E-01 2.70E-02	**2.40E-01** **1.04E-02**
$f_{14}$	Mean SD	7.26E+01 1.44E+01	5.14E+00 2.71E+00	2.39E-01 2.98E-02	5.59E+00 1.01E+01	4.00E-01 5.41E-02	3.75E-01 1.33E-01	2.91E-01 2.05E-02	**1.88E-01** **2.03E-02**
$f_{15}$	Mean SD	1.37E+03 6.14E+02	8.27E+02 4.27E+02	1.38E+01 3.13E+00	2.98E+03 1.75E+03	2.07E+01 1.87E+00	1.33E+01 2.13E+00	1.91E+01 1.31E+01	**6.21E+00** **3.82E+00**
$f_{16}$	Mean SD	1.37E+01 2.70E-01	1.29E+01 2.28E-01	1.13E+01 3.30E-01	4.48E+01 8.60E-01	1.26E+01 2.75E-01	1.04E+01 3.92E-01	1.30E+01 8.17E-01	**1.01E+01** **2.03E-01**
$f_{17}$	Mean SD	2.30E+07 6.61E+06	4.31E+06 1.72E+06	5.98E+06 3.90E+06	4.50E+07 1.46E+07	**1.64E+04** **9.04E+03**	2.31E+05 1.72E+05	4.00E+05 2.63E+05	3.12E+05 2.23E+05
$f_{18}$	Mean SD	5.36E+02 3.37E+02	1.12E+07 6.16E+06	7.51E+03 7.16E+03	8.16E+06 1.06E+07	5.43E+03 2.73E+03	3.07E+03 2.80E+03	2.22E+02 2.96E+02	**2.04E+02** **2.90E+02**
$f_{19}$	Mean SD	1.73E+02 3.09E+01	4.87E+01 1.61E+01	1.81E+01 7.10E+00	2.32E+02 4.93E+01	1.30E+01 8.47E-01	1.02E+01 1.57E+01	2.22E+01 3.14E+01	**1.00E+01** **1.12E+01**
$f_{20}$	Mean SD	2.54E+05 1.26E+05	2.06E+04 9.05E+03	1.49E+04 9.14E+03	1.26E+05 3.31E+04	**1.13E+02** **1.98E+01**	8.31E+03 4.62E+03	1.35E+04 7.32E+03	5.45E+03 3.32E+03
$f_{21}$	Mean SD	1.01E+07 4.52E+06	7.15E+05 3.96E+05	8.89E+05 6.78E+05	1.88E+07 6.27E+06	**2.67E+03** **4.83E+02**	1.41E+05 9.40E+04	2.04E+05 1.04E+05	1.55E+05 8.36E+04
$f_{22}$	Mean SD	1.27E+03 3.24E+02	4.98E+02 1.27E+02	4.55E+02 1.21E+02	3.05E+03 4.81E+02	4.89E+02 9.56E+01	3.11E+02 2.20E+02	5.04E+02 1.93E+02	**1.57E+02** **4.33E+01**
$f_{23}$	Mean SD	2.66E+02 9.55E+01	3.37E+02 4.88E+00	3.19E+02 2.36E+00	4.26E+02 1.53E+01	3.16E+02 2.68E-01	3.15E+02 9.01E-12	3.16E+02 9.29E-01	**2.03E+02** **7.93E-01**
$f_{24}$	Mean SD	2.15E+02 8.30E+00	2.70E+02 3.52E+00	2.30E+02 1.72E+00	4.03E+02 8.92E+00	2.41E+02 3.53E+00	2.30E+02 5.26E+00	2.36E+02 5.65E+00	**1.29E+02** **5.58E+00**
$f_{25}$	Mean SD	2.05E+02 2.49E+00	2.22E+02 2.80E+00	2.12E+02 1.59E+00	3.35E+02 2.00E+01	2.08E+02 1.24E+00	2.05E+02 2.43E+00	2.15E+02 4.47E+00	**2.05E+02** **1.76E+00**
$f_{26}$	Mean SD	1.90E+02 2.67E+01	1.01E+02 5.94E-01	1.00E+02 5.90E-02	2.05E+02 1.55E+00	1.01E+02 8.04E-02	1.17E+02 4.88E+01	1.00E+02 7.45E-02	**1.00E+02** **2.32E-02**
$f_{27}$	Mean SD	1.74E+03 3.65E+02	5.00E+02 3.04E+01	4.84E+02 1.51E+01	3.55E+03 1.85E+02	5.07E+02 1.28E+02	4.42E+02 6.42E+01	6.87E+02 2.05E+02	**4.03E+02** **2.05E+02**
$f_{28}$	Mean SD	2.31E+03 9.41E+02	1.61E+03 1.80E+02	1.18E+03 1.46E+02	1.99E+04 3.25E+03	1.14E+03 2.19E+01	**9.05E+02** **5.31E+01**	1.97E+03 6.94E+02	1.18E+03 4.34E+02
$f_{29}$	Mean SD	3.30E+07 1.12E+08	9.25E+04 3.55E+04	1.71E+03 4.92E+02	8.13E+05 6.96E+05	7.30E+03 3.08E+03	1.30E+03 4.04E+02	1.24E+03 3.92E+02	**9.28E+02** **2.88E+02**
$f_{30}$	Mean SD	1.79E+06 6.33E+05	3.02E+04 8.06E+03	8.24E+03 3.90E+03	7.73E+05 2.26E+05	5.57E+03 1.19E+03	2.73E+03 9.21E+02	2.17E+03 1.57E+03	**2.00E+03** **1.02E+03**

**Table 11:** The mean minimization value and standard deviation (SD) over 30 trials with D = 50.

Functions	Meas.	GSA	CLPSO	ABC	GSO	CoDE	EFO	CSS	CSSRank
$f_{1}$	Mean SD	4.37E+08 2.51E+08	1.48E+08 2.57E+07	2.81E+07 1.01E+07	2.39E+07 6.94E+06	1.21E+07 4.48E+06	**1.52E+06** **5.46E+05**	7.12E+07 4.74E+07	1.89E+07 1.98E+07
$f_{2}$	Mean SD	3.04E+10 4.95E+09	6.81E+09 1.12E+09	2.88E+04 4.11E+04	4.88E+07 2.58E+07	1.89E+07 9.45E+06	6.83E+03 7.04E+03	7.35E+03 7.77E+03	**3.05E+03** **2.91E+03**
$f_{3}$	Mean SD	1.52E+05 7.53E+03	9.86E+04 1.68E+04	1.06E+04 3.66E+03	2.00E+04 6.02E+03	**4.16E+03** **1.89E+03**	3.18E+04 1.39E+04	3.77E+04 3.12E+04	2.89E+04 1.88E+04
$f_{4}$	Mean SD	4.36E+03 8.38E+02	9.77E+02 1.39E+02	1.59E+02 2.76E+01	2.92E+02 5.23E+01	1.44E+02 1.55E+01	9.44E+01 2.91E+01	2.03E+02 1.05E+02	**6.84E+01** **4.44E+02**
$f_{5}$	Mean SD	**2.00E+01** **8.83E-05**	2.11E+01 4.87E-02	2.04E+01 3.53E-02	2.00E+01 2.90E-02	2.10E+01 6.56E-02	2.00E+01 2.67E-02	2.00E+01 8.50E-02	2.00E+01 9.33E-02
$f_{6}$	Mean SD	5.57E+01 3.12E+00	5.08E+01 2.46E+00	3.78E+01 2.65E+00	4.72E+01 4.07E+00	5.57E+01 2.67E+00	**1.60E+01** **2.31E+00**	2.99E+01 4.98E+00	1.60E+01 3.33E+00
$f_{7}$	Mean SD	2.85E+02 4.55E+01	6.32E+01 8.63E+00	5.72E-01 1.36E-01	1.81E+00 5.33E-01	1.20E+00 7.20E-02	2.66E-02 5.17E-02	2.03E-02 2.13E-02	**7.09E-03** **5.21E-03**
$f_{8}$	Mean SD	2.81E+02 1.96E+01	2.92E+02 1.87E+01	1.26E+01 1.74E+00	7.70E+01 1.60E+01	2.30E+02 1.45E+01	**1.37E+00** **1.42E+00**	9.25E+01 3.04E+01	6.91E+01 6.09E+01
$f_{9}$	Mean SD	3.64E+02 3.12E+01	4.73E+02 2.13E+01	2.58E+02 2.83E+01	3.09E+02 4.95E+01	3.80E+02 1.89E+01	1.91E+02 1.41E+02	1.91E+02 8.19E+01	**1.42E+02** **3.85E+01**
$f_{10}$	Mean SD	7.68E+03 7.20E+02	7.62E+03 5.19E+02	2.29E+02 1.07E+02	1.01E+03 3.73E+02	7.26E+03 3.84E+02	**4.70E+00** **2.04E+00**	3.25E+03 6.59E+02	2.15E+03 3.08E+02
$f_{11}$	Mean SD	8.54E+03 7.53E+02	1.14E+04 5.09E+02	5.74E+03 3.27E+02	6.79E+03 9.11E+02	1.21E+04 4.27E+02	1.36E+04 6.24E+02	**3.17E+03** **1.39E+02**	5.33E+03 2.06E+02
$f_{12}$	Mean SD	**1.32E-02** **1.06E-02**	2.67E+00 3.28E-01	4.71E-01 5.73E-02	6.03E-01 1.91E-01	2.47E+00 2.74E-01	3.94E+00 3.38E-01	7.17E-01 9.66E-01	2.13E-01 4.69E-01
$f_{13}$	Mean SD	3.79E+00 2.45E-01	7.61E-01 8.47E-02	4.51E-01 4.11E-02	5.59E-01 6.64E-02	6.53E-01 6.56E-02	3.51E-01 6.35E-02	3.62E-01 1.16E-01	**3.50E-01 ** **5.79E-02**
$f_{14}$	Mean SD	6.16E+01 1.46E+01	1.60E+01 3.71E+00	2.98E-01 2.50E-02	3.09E-01 2.80E-02	4.31E-01 8.50E-02	4.20E-01 2.15E-01	3.82E-01 7.44E-02	**2.23E-01 ** **3.26E-02**
$f_{15}$	Mean SD	2.07E+04 1.16E+04	3.31E+03 1.93E+03	3.14E+01 6.02E+00	1.47E+02 3.90E+01	3.78E+01 2.26E+00	2.47E+01 7.71E+00	4.69E+01 2.12E+01	**2.44E+01 ** **5.02E+00**
$f_{16}$	Mean SD	2.26E+01 4.78E-01	2.24E+01 2.33E-01	1.97E+01 4.02E-01	2.28E+01 3.05E-01	2.28E+01 3.26E-01	2.22E+01 3.40E-01	**2.08E+01 ** **8.32E-01**	2.24E+01 3.68E-01
$f_{17}$	Mean SD	3.49E+07 1.48E+07	1.77E+07 4.74E+06	1.01E+07 4.96E+06	4.00E+06 1.42E+06	**1.81E+05** **1.24E+05**	2.84E+05 1.05E+05	2.38E+06 2.63E+06	7.60E+05 6.45E+05
$f_{18}$	Mean SD	6.93E+08 8.46E+08	2.51E+07 8.22E+06	9.92E+03 9.94E+03	1.28E+03 9.26E+02	3.62E+03 2.31E+03	8.97E+02 9.37E+02	9.56E+02 1.06E+03	**5.25E+02 ** **3.21E+02**
$f_{19}$	Mean SD	2.02E+02 3.38E+01	9.23E+01 1.19E+01	**3.33E+01** **1.06E+01**	5.37E+01 3.36E+01	3.62E+01 1.08E+01	5.84E+01 2.17E+01	5.10E+01 3.59E+01	4.94E+01 3.30E+01
$f_{20}$	Mean SD	1.07E+05 4.22E+04	5.17E+04 1.06E+04	3.96E+04 1.29E+04	1.94E+04 1.11E+04	**5.04E+02** **3.17E+02**	1.10E+04 6.66E+03	1.78E+04 6.18E+03	1.40E+04 3.49E+03
$f_{21}$	Mean SD	4.19E+06 1.42E+06	5.71E+06 2.15E+06	7.30E+06 4.36E+06	3.36E+06 1.65E+06	**2.12E+04** **1.61E+04**	2.72E+05 1.51E+05	6.26E+05 5.25E+05	5.24E+05 2.07E+05
$f_{22}$	Mean SD	2.45E+03 7.30E+02	1.36E+03 1.71E+02	1.14E+03 1.89E+02	1.48E+03 3.27E+02	1.44E+03 1.59E+02	1.12E+03 2.86E+02	1.14E+03 2.40E+02	**8.39E+02 ** **1.35E+02**
$f_{23}$	Mean SD	2.14E+02 7.68E+01	3.90E+02 8.19E+00	3.57E+02 7.30E+00	3.54E+02 1.97E+00	3.55E+02 1.77E-01	3.44E+02 3.25E-10	3.44E+02 3.00E-01	**2.11E+02 ** **5.39E-01**
$f_{24}$	Mean SD	2.47E+02 1.12E+01	3.39E+02 6.72E+00	2.71E+02 1.78E+00	2.70E+02 7.39E+00	2.83E+02 1.80E+00	2.69E+02 6.14E+00	2.62E+02 8.92E+00	**2.00E+02 ** **6.67E+00**
$f_{25}$	Mean SD	**2.03E+02** **4.02E+00**	2.46E+02 5.30E+00	2.22E+02 2.80E+00	2.48E+02 8.18E+00	2.18E+02 1.94E+00	2.18E+02 3.58E+00	2.29E+2 4.97E+00	2.05E+02 3.61E+00
$f_{26}$	Mean SD	2.00E+02 8.47E-02	1.10E+02 2.83E+01	1.01E+02 6.75E-02	1.94E+02 2.55E+01	1.04E+02 1.82E+01	1.62E+02 5.97E+01	1.10E+02 3.15E-01	**1.00E+02 ** **1.90E-01**
$f_{27}$	Mean SD	3.29E+03 7.49E+02	1.33E+03 3.66E+02	1.08E+03 3.78E+02	1.63E+03 9.43E+01	1.28E+03 1.47E+02	7.98E+02 6.71E+01	1.24E+03 1.30E+02	**5.44E+02 ** **4.88E+02**
$f_{28}$	Mean SD	5.71E+03 1.11E+03	3.02E+03 4.20E+02	2.15E+03 3.42E+02	7.04E+03 1.17E+03	1.92E+03 1.26E+02	1.55E+03 2.22E+02	2.54E+03 8.73E+02	**1.09E+03** **7.14E+02**
$f_{29}$	Mean SD	**2.00E+02** **7.50E-02**	4.10E+05 1.64E+05	3.32E+03 1.46E+03	5.43E+03 2.32E+03	2.00E+04 7.15E+03	1.91E+03 5.37E+02	1.83E+03 1.42E+03	2.00E+03 1.11E+03
$f_{30}$	Mean SD	4.97E+06 5.07E+06	6.00E+04 1.52E+04	1.61E+04 4.10E+03	5.17E+04 1.36E+04	1.97E+04 2.00E+03	1.24E+04 2.45E+03	4.46E+04 6.21E+03	**9.31E+03** **3.49E+03**

On 50D problems, CSSRank found the best result for the 15 problems compared to the other algorithms. In comparison, EFO, CODE, and GSA performed best for four problems, standard CSS found the best result two times, and ABC reached the best result for just one function. However, the CLPSO and GSO algorithms did not outperform the other methods. Additionally, it is noteworthy that the standard deviation for CSSRank in the functions where it achieved the best results is significantly lower than that of other algorithms, indicating its stability and consistency in obtaining optimal solutions.

**Fig. 10:**
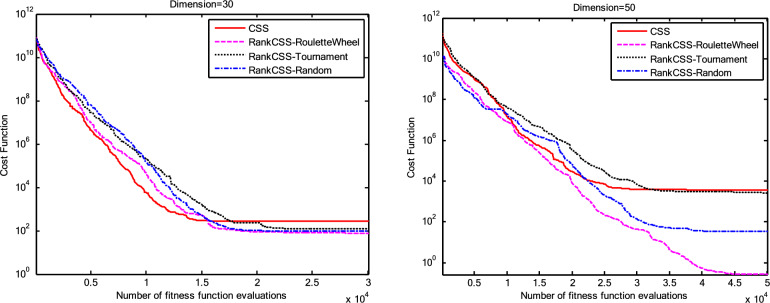
Convergence curves of best value for unimodal function 2 (CEC 2014).

**Fig. 11:**
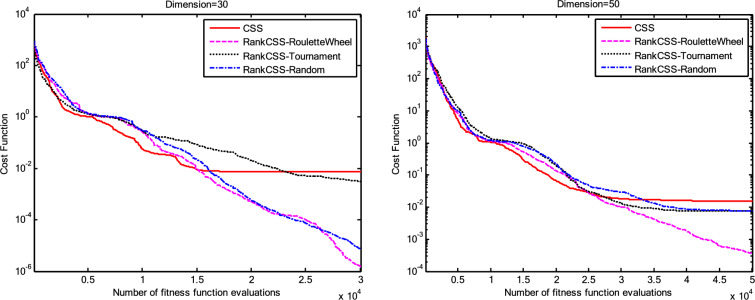
Convergence curves of best value for the multimodal function 7 (CEC 2014).

**Fig. 12:**
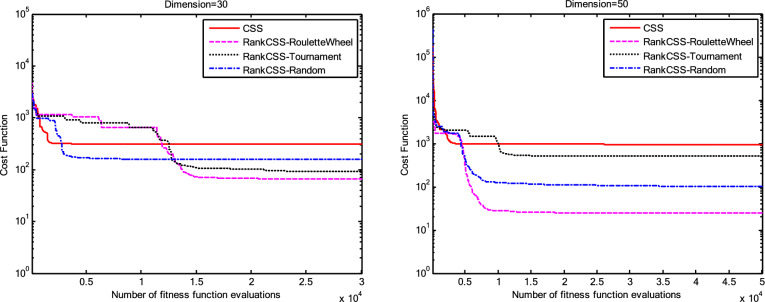
Convergence curves of best value for the hybrid function 22 (CEC 2014).

**Fig. 13:**
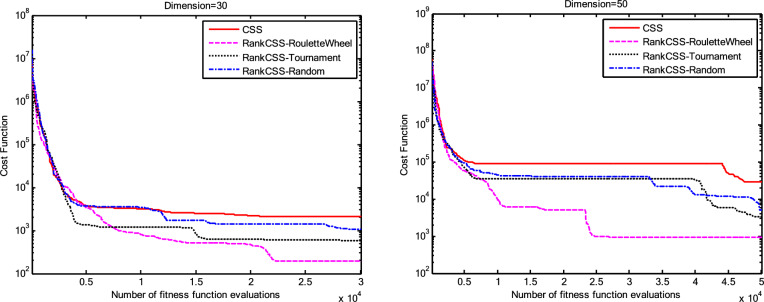
Convergence curves of best value for the composition function 30 (CEC 2014).

The results of the Friedman test ranks presented in Tables 12 and 13 confirm that CSSRank has a lower summary rank compared to other algorithms, indicating its superior performance in both dimensions. Figs. 10,11,12,13 depict the convergence rate versus the number of fitness function evaluations of the best solution obtained using different selection methods in 30 runs. For each benchmark function class, one function is selected as the representative (Function No. 2, 7, 22, and 30). It is evident that the roulette wheel method exhibits better convergence than the other selection methods.

**Table 12:** Mean Friedman ranks of error for the CEC2014 benchmark functions (D=30).

Functions	GSA	CLPSO	ABC	GSO	CoDE	EFO	CSS	CSSRank
$f_{1}$	7.90	6.07	4.53	7.03	2.57	1.27	3.60	3.03
$f_{2}$	7.63	6.00	3.97	7.37	5.00	1.73	2.47	1.83
$f_{3}$	7.17	6.00	2.47	7.80	1.40	2.40	4.93	3.83
$f_{4}$	7.10	6.00	3.57	7.90	4.97	2.10	2.83	1.53
$f_{5}$	2.53	8.00	5.50	5.50	7.00	2.43	2.63	2.40
$f_{6}$	6.33	5.43	4.00	8.00	6.23	2.70	1.83	1.47
$f_{7}$	7.97	6.00	4.00	7.03	5.00	2.63	1.70	1.67
$f_{8}$	6.13	6.87	2.00	8.00	4.97	1.00	3.80	3.23
$f_{9}$	4.57	7.00	3.37	8.00	5.70	4.30	1.93	1.13
$f_{10}$	6.13	6.70	1.90	8.00	5.17	1.10	3.70	3.30
$f_{11}$	4.00	5.70	3.00	8.00	5.37	6.93	1.23	1.77
$f_{12}$	1.93	7.07	4.00	5.03	6.03	7.87	2.37	1.70
$f_{13}$	8.00	6.67	3.83	5.50	5.83	2.63	2.30	1.23
$f_{14}$	8.00	6.70	2.50	4.47	5.00	4.20	3.53	1.60
$f_{15}$	6.93	6.40	3.00	7.67	4.57	2.83	3.23	1.37
$f_{16}$	6.77	5.37	3.03	8.00	4.43	1.83	5.37	1.20
$f_{17}$	7.00	5.40	5.47	8.00	1.03	2.77	3.33	3.00
$f_{18}$	2.90	7.77	4.93	7.23	5.17	4.33	1.80	1.87
$f_{19}$	7.17	5.77	3.93	7.83	3.20	2.20	3.37	2.53
$f_{20}$	7.70	5.23	4.47	7.13	1.07	3.40	4.33	2.67
$f_{21}$	7.07	5.17	4.90	7.87	1.13	3.03	3.90	2.93
$f_{22}$	6.93	4.43	3.77	8.00	4.37	2.63	4.57	1.30
$f_{23}$	3.23	6.80	5.37	8.00	4.23	2.87	4.20	1.30
$f_{24}$	2.10	7.00	3.60	8.00	5.73	3.67	4.90	1.00
$f_{25}$	2.17	6.97	5.20	8.00	3.80	2.10	5.67	2.10
$f_{26}$	7.17	4.83	2.40	7.67	4.93	4.00	2.53	2.47
$f_{27}$	7.00	3.67	3.13	8.00	3.50	2.40	5.47	2.83
$f_{28}$	5.80	5.43	3.23	8.00	3.10	1.50	5.53	3.40
$f_{29}$	7.80	6.13	3.47	7.07	4.87	2.67	2.47	1.53
$f_{30}$	7.93	6.00	4.43	7.07	4.20	2.40	2.00	1.97
**Sum**	**181.07**	**182.57**	**112.97**	**221.17**	**129.57**	**87.93**	**101.53**	**63.20**

**Table 13:** Mean Friedman ranks of error for the CEC2014 benchmark functions (D=50).

Functions	GSA	CLPSO	ABC	GSO	CoDE	EFO	CSS	CSSRank
$f_{1}$	7.63	7.10	4.70	4.20	2.83	1.10	5.27	3.17
$f_{2}$	8.00	7.00	2.97	5.83	5.17	2.67	2.33	2.03
$f_{3}$	8.00	6.90	2.57	3.77	1.40	4.63	4.50	4.23
$f_{4}$	8.00	6.97	3.90	5.70	3.57	2.20	4.47	1.20
$f_{5}$	3.13	7.97	6.00	3.10	7.03	3.03	2.80	2.93
$f_{6}$	7.23	5.90	4.00	5.30	7.53	1.53	3.03	1.47
$f_{7}$	8.00	7.00	4.00	5.87	5.13	2.13	2.03	1.83
$f_{8}$	7.33	7.63	2.23	3.90	6.03	1.13	4.37	3.37
$f_{9}$	5.90	7.93	3.60	4.73	6.53	2.87	2.67	1.77
$f_{10}$	7.33	6.93	2.03	2.97	6.73	1.00	4.93	4.07
$f_{11}$	4.90	6.07	2.90	4.07	6.93	8.00	1.00	2.13
$f_{12}$	1.33	6.70	3.77	4.27	6.17	7.93	3.57	2.27
$f_{13}$	8.00	6.77	3.73	5.03	6.03	2.13	2.47	1.83
$f_{14}$	8.00	7.00	2.83	3.13	4.97	4.33	4.47	1.27
$f_{15}$	7.93	7.07	2.83	6.00	4.10	1.97	4.17	1.93
$f_{16}$	5.90	4.73	1.10	6.73	6.63	3.87	2.13	4.90
$f_{17}$	7.87	7.03	5.87	4.90	1.63	2.13	3.53	3.03
$f_{18}$	7.93	7.07	4.83	3.40	4.97	2.67	2.67	2.47
$f_{19}$	8.00	6.50	2.77	4.10	2.70	4.40	3.93	3.60
$f_{20}$	7.60	6.70	6.23	4.23	1.03	2.90	4.03	3.27
$f_{21}$	6.33	6.93	7.00	5.57	1.00	2.67	3.10	3.40
$f_{22}$	7.43	5.00	3.33	5.77	5.97	3.50	3.47	1.53
$f_{23}$	1.67	7.97	6.10	5.57	6.17	3.50	3.50	1.53
$f_{24}$	2.40	8.00	5.00	4.80	6.93	4.53	3.33	1.00
$f_{25}$	1.30	7.50	4.83	7.50	3.57	3.73	5.87	1.70
$f_{26}$	7.30	3.87	2.83	7.10	3.20	5.70	4.20	1.80
$f_{27}$	8.00	4.90	3.73	6.67	4.60	1.93	4.37	1.80
$f_{28}$	7.20	5.70	3.87	7.80	3.37	2.13	4.23	1.70
$f_{29}$	1.13	8.00	4.53	5.73	6.80	3.30	3.03	3.47
$f_{30}$	7.83	6.63	2.77	5.90	3.77	2.13	5.63	1.33
**Sum**	**188.63**	**201.47**	**116.87**	**153.63**	**142.50**	**95.77**	**109.10**	**72.03**

### CEC 2024 problems

To compare the developed CSSRank with several state-of-the-art optimization methods, we utilized the problems from the CEC 2024 competition in this subsection. The results were compared with the top 6 winners of the competition. There are 29 problems in total, and all problems have a dimensionality of 30, as recommended by the CEC 2024 committee.

The maximum number of function evaluations is limited to 300,000, and 25 independent runs were conducted for each algorithm, with different initializations. The comparison process is based on the final results for cases where algorithms do not reach the target optimum point (predefined threshold values), and the number of required function evaluations for cases where the optimum solution is reached. A smaller number of function evaluations is better, and for those that do not reach the final solution, a smaller objective function value is preferred. Based on this rule, all 25 runs for all algorithms are ranked. The score for the *i*th run of algorithm j can be calculated as

16 $${Score}_{i,j}=\frac{Nruns\left(Nruns-1\right)}{2}-{Rank}_{i,j}$$

where *Nruns* is the number of runs for each algorithm (25).

The winning algorithms of this competition, which represent the most powerful methods for numerical optimization, include:

- Success rate-based adaptive differential evolution (L-SRTDE),
- Reconstructed differential evolution (RDE)
- Block evolutionary algorithm(BEA)
- Multi-operator ensemble LSHADE with restart and local search mechanisms (mLSHADE)
- Progressive archive in adaptive jSO Algorithm (jSO),

A modified EACOP (iEACOP). Tables 14 and 15 present the scores and corresponding ranks for these algorithms, as well as for CSSRank. Based on the results, it is evident that L-SRTDE and RDE are superior compared to the other methods, which perform similarly overall. If we rank the performance of the new algorithm, it places 5th out of 7. However, the difference between the third and the last algorithm is very small. This indicates that, although the developed algorithm is not specifically tailored for these particular problems, it still performs comparably to the winning methods of the competition. In conclusion, the ability of CSSRank to rank closely with the top-performing algorithms in the competition highlights its robustness and versatility.

**Table 14:** Score obtained for state-of-the-art algorithms based on 25 independent runs for the CEC2024 functions.

Functions	L-SRDE	RDE	BEA	mLSHADE	jSO	IEACOP	CSSRank
$f_{1}$	2275	2900	625	955	1625	3750	995
$f_{2}$	3750	1875	195	430	3125	2500	1250
$f_{3}$	2308	2455	0	1250	2737	3750	625
$f_{4}$	3588	1776	1113	2852	1310	114	2372
$f_{5}$	2358	3100	3750	0	1654	1466	797
$f_{6}$	1605	1234	3750	3125	869	42	2500
$f_{7}$	3473	2201	2350	47	1763	804	2487
$f_{8}$	2617	3325	2856	31	1975	1071	1250
$f_{9}$	1875	913	3750	3125	924	38	2500
$f_{10}$	2513	3112	520	105	1875	3750	1250
$f_{11}$	1389	2049	3524	3351	894	0	1918
$f_{12}$	3400	2753	1481	323	2075	1728	1365
$f_{13}$	3055	3576	0	1513	2375	1899	707
$f_{14}$	2278	2768	0	3532	1250	2672	625
$f_{15}$	3092	1838	639	3725	1057	614	2160
$f_{16}$	3451	1791	2019	1262	1145	1032	2425
$f_{17}$	3290	3585	1190	823	2454	33	1750
$f_{18}$	2457	3169	576	1815	1154	3429	525
$f_{19}$	0	2407	744	3468	2070	1131	3305
$f_{20}$	1927	1275	3369	3147	699	30	2678
$f_{21}$	2500	3125	20	605	1875	3750	1250
$f_{22}$	2616	2131	425	1075	2779	3724	375
$f_{23}$	1854	1235	3750	3078	661	0	2547
$f_{24}$	2438	3252	10	615	1941	3619	1250
$f_{25}$	2025	984	3750	3125	559	632	2050
$f_{26}$	1250	2487	3103	0	2033	3627	625
$f_{27}$	3194	2382	179	446	2113	3361	1450
$f_{28}$	3744	1617	1734	3041	717	2	2270
$f_{29}$	2703	1250	3697	2975	625	0	1875
**Sum**	**73025**	**66565**	**49119**	**49839**	**46333**	**48568**	**47176**

**Table 15:** Rank obtained for state-of-the-art algorithms based on 25 indepenentruns for the CEC2024 functions.

Functions	L-SRDE	RDE	BEA	mLSHADE	jSO	IEACOP	CSSRank
$f_{1}$	_3_	_2_	_7_	_6_	_4_	_1_	_5_
$f_{2}$	_1_	_4_	_7_	_6_	_2_	_3_	_5_
$f_{3}$	_4_	_3_	_7_	_5_	_2_	_1_	_6_
$f_{4}$	_1_	_4_	_6_	_2_	_5_	_7_	_3_
$f_{5}$	_3_	_2_	_1_	_7_	_4_	_5_	_6_
$f_{6}$	_4_	_5_	_1_	_2_	_6_	_7_	_3_
$f_{7}$	_1_	_4_	_3_	_7_	_5_	_6_	_2_
$f_{8}$	_3_	_1_	_2_	_7_	_4_	_6_	_5_
$f_{9}$	_4_	_6_	_1_	_2_	_5_	_7_	_3_
$f_{10}$	_3_	_2_	_6_	_7_	_4_	_1_	_5_
$f_{11}$	_5_	_3_	_1_	_2_	_6_	_7_	_4_
$f_{12}$	_1_	_2_	_5_	_7_	_3_	_4_	_6_
$f_{13}$	_2_	_1_	_7_	_5_	_3_	_4_	_6_
$f_{14}$	_4_	_2_	_7_	_1_	_5_	_3_	_6_
$f_{15}$	_2_	_4_	_6_	_1_	_5_	_7_	_3_
$f_{16}$	_1_	_4_	_3_	_5_	_6_	_7_	_2_
$f_{17}$	_2_	_1_	_5_	_6_	_3_	_7_	_4_
$f_{18}$	_3_	_2_	_6_	_4_	_5_	_1_	_7_
$f_{19}$	_7_	_3_	_6_	_1_	_4_	_5_	_2_
$f_{20}$	_4_	_5_	_1_	_2_	_6_	_7_	_3_
$f_{21}$	_3_	_2_	_7_	_6_	_4_	_1_	_5_
$f_{22}$	_3_	_4_	_6_	_5_	_2_	_1_	_7_
$f_{23}$	_4_	_5_	_1_	_2_	_6_	_7_	_3_
$f_{24}$	_3_	_2_	_7_	_6_	_4_	_1_	_5_
$f_{25}$	_4_	_5_	_1_	_2_	_7_	_6_	_3_
$f_{26}$	_5_	_3_	_2_	_7_	_4_	_1_	_6_
$f_{27}$	_2_	_3_	_7_	_6_	_4_	_1_	_5_
$f_{28}$	_1_	_5_	_4_	_2_	_6_	_7_	_3_
$f_{29}$	_3_	_5_	_1_	_2_	_6_	_7_	_4_
**Sum**	_**86**_	_**94**_	_**124**_	_**123**_	_**130**_	_**128**_	_**127**_

## Application of CSSRank to data clustering

Cluster analysis, or clustering, is a fundamental technique utilized across various disciplines, including pattern recognition, data mining, information retrieval, and image analysis^35^. The primary goal of cluster analysis is to categorize a collection of items into groups or clusters, where items within the same cluster share more similarities with each other than with those in other clusters. This process is driven by predefined criteria, which help identify patterns and structures within the data, facilitating further analysis and interpretation^36^.Over the years, many algorithms have been proposed to solve clustering problems. In particular, the K-means algorithm is the most popular and widely used partition-based algorithm^37^. Although this algorithm has the advantages of being fast, easy, and simple to implement, it has some disadvantages. Specifically, the initial cluster centers can highly affect the performance of the K-means algorithm, and because of its non-convex objective function, the solutions might get stuck in local minima so that a local minimum is obtained instead of the global one^38^. Therefore, several meta-heuristic optimization algorithms have been developed to overcome these drawbacks. Particle swarm optimization (PSO)^38^, ant colony optimization (ACO)^39^, artificial bee colony (ABC)^40^, differential evolution (DE)^41^, genetic algorithm (GA)^42^, big bang- big crunch (BB-BC)^43^, Bat Algorithm^44^, and K-means^45^ are examples of such algorithms that have been employed for clustering tasks.

### Clustering problem and K-means algorithm

Let $X = \left[ {X_{1} ,X_{2} ,...,X_{N} } \right]$, where $X_{i} \in R^{D}$ is a dataset of *N* data objects to be clustered, and $C = \left[ {C_{1} ,C_{2} ,...,C_{K} } \right]$ is a set of *K* clusters. In the *K*-means algorithm, each data in set *X* will be assigned to one of the *K* clusters in such a way that minimizes the intra-cluster distance. So, the objective function is defined by the sum of the squared Euclidean distance between each object *X*_*i*_ and the cluster center *M*_*j*_ as:

17 $$\begin{gathered} F\left( {M,X} \right) = \sum\limits_{i = 1}^{N} {Min} \left\{ {\left\| {X_{i} - M_{j} } \right\|^{2} } \right\},\quad j = 1,2,...,K\quad \quad \quad \quad \quad \hfill \\ Also \hfill \\ C_{j} \ne \emptyset ,{\kern 1pt} \;\forall j\; \in \;\left\{ {1,2,...,K} \right\} \hfill \\ C_{i} \cap C_{j} = \emptyset ,\;\forall i \ne j\;and\;\forall i,j \in \left\{ {1,2,...K} \right\} \hfill \\ \bigcup\limits_{j = 1}^{k} {Cj} = X \hfill \\ \end{gathered}$$

### UCI benchmark datasets

After reviewing various metaheuristic algorithms applied to data clustering problems and the datasets commonly used in the literature, it is observed that Iris, Wine, Glass, CMC, Breast Cancer, and Vowel are among the most frequently employed benchmark datasets for evaluating clustering algorithms^39^. These datasets were therefore selected from the UCI Machine Learning Repository to assess the performance of the proposed CSSRank algorithm across a diverse range of real-world clustering scenarios. These datasets offer varying levels of complexity, dimensionality, and cluster distributions, ranging from balanced to highly imbalanced class sizes, and originate from different application domains. In all cases, the number of clusters *K* and the number of data objects are known in advance. The objective is to determine the optimal cluster centers in such a way that minimizes the clustering objective function. A brief overview of the datasets is provided below^39^:

- The breast cancer Wisconsin dataset (K=2, *N*_*data*_=683, *D*=9) contains two categories of breast cancer: malignant (444 instances) and benign (239 instances), with nine features, which include clump thickness, cell size uniformity, cell shape uniformity, marginal adhesion, single epithelial cell size, bare nuclei, bland chromatin, normal nucleoli, and mitoses.
- The contraceptive Method Choice dataset (*K*=3, *N*_*data*_=1473, *D*=9) contains information about married women who were not pregnant with three choices of current contraceptive method: no use (629 instances), long-term method (334 instances) and short-term method (510 instances) and nine features, which include age, wife’s education, husband’s education, number of children ever born, wife’s religion, wife’s current work, husband’s occupation, standard-of-living index, and media exposure.
- The Iris dataset (*K*=3, *N*_*data*_=150, *D*=4) contains three varieties of iris flowers: setosa, versicolour, and virginica. For each class, 50 instances with four features (sepal length, sepal width, petal length, and petal width) were collected.
- Glass dataset (*K*=6, *N*_*data*_=214, *D*=9) contains six different types of glass: building windows float processed (70 instances), building windows non-float processed (76 instances), vehicle windows float processed (17 instances), containers (13 instances), table-ware (9 instances), and headlamps (29 instances) with nine features (refractive index, sodium, magnesium, aluminum, silicon, potassium, calcium, barium, and iron).
- The vowel dataset (*K*=6, *N*_*data*_=871, *D*=3) contains 871 data instances of Indian Telugu vowel sounds with six vowel classes: d (72 instances), a (89 instances), i (172 instances), u (151 instances), e (207 instances) and o (180 instances) and three features (first, second, and third vowel frequencies).
- The wine dataset (*K*=3, *N*_*data*_=178, *D*=13) contains information about the chemical analysis of wine in the same region of Italy but derived from three different cultivators: class 1 (59 instances), class 2 (71 instances), and class 3 (48 instances). Thirteen features of this dataset are alcohol, malic acid, ash, alkalinity of ash, magnesium, total phenols, flavanoids, nonflavanoid phenols, proanthocyanins, color intensity, hue, OD280/OD315 of diluted wines, and proline.

The description summary of the main characteristics of these datasets is given in Table 16.

**Table 16:** Summarized characteristics of the UCI benchmark datasets.

**Dataset**	**Domain**	**Instances**	**Features**	**Clusters**	**Updated notes**
**CANCER**	Clinical diagnostics	683	9	2	Medium dimensional, binary classification, moderately imbalanced
**CMC**	Demographics	1473	9	3	Balanced multi-class, social survey data, semantic heterogeneity
**IRIS**	Plant biology	150	4	3	Balanced, low dimensional, well-separated clusters
**GLASS**	Materials	214	9	6	Highly imbalanced, overlapping clusters, very small clusters
**VOWEL**	Acoustic phonetics	871	3	6	Overlapping classes, moderate imbalance, phonetic frequencies
**WINE**	Chemistry/agriculture	178	13	3	High dimensional, scale heterogeneity, moderate imbalance

### Results

In this study, two evaluation criteria were employed to assess the clustering performance of the algorithms: total intra-cluster distance and clustering accuracy^40,41^. The accuracy metric reflects how well the predicted cluster labels match the true labels and is computed using a mapping function that aligns predicted clusters with ground truth class labels. Both the standard CSS and the proposed CSSRank algorithms were executed independently 30 times using the same experimental setup described in Section "Rank-based charged system search algorithm", with the maximum number of iterations set to 300.

Table 17 presents the results for intra-cluster distance, reported in terms of the best, average, and standard deviation values across 30 runs. The results indicate that CSSRank consistently outperforms the standard CSS algorithm across all selection strategies. Among them, the roulette wheel selection method yielded the lowest average intra-cluster distances and the smallest standard deviations, highlighting its robustness and effectiveness in clustering tasks. Table 18 reports the clustering accuracy results for both CSS and CSSRank. The proposed algorithm achieved higher accuracy across most datasets. Specifically, CSSRank with roulette wheel selection outperformed other variants on all datasets except for CMC and Breast Cancer, where tournament selection led to better accuracy. These results demonstrate the adaptability of CSSRank and the strong general performance of the roulette wheel-based variant in clustering tasks.

**Table 17:** Comparison of the intra-cluster distance parameter for the results of the CSS and CSSRank algorithms.

Dataset	Criteria	CSS	CSSRank		
			Roulette wheel	Tournament	Random
Iris	Best Average Std	96.6911 96.7399 0.0180	96.6554 96.6554 3.10e-10	96.6554 96.6564 0.00333	**96.6554** **96.6554** **2.51e-14**
Glass	Best Average Std	217. 6151 237.1002 12.1976	**210.4309** **215.0769** **3.8387**	210.4287 217.0526 9.4793	210.4508 219.9011 9.3904
Wine	Best Average Std	16299.3063 16303.3551 1.6281	**16292.1846** **16292.9282** **0. 6559**	16292.1846 16293.1023 0.777	16292.1848 16293.5032 0.8366
Vowel	Best Average Std	149288.5993 151772.0473 1338	**148967.2408** **149234.5179** **289.4320**	149041.1312 149331.7624 441.9261	148967.2408 149537.5068 637.9975
CMC	Best Average Std	5693.0430 5697.4096 2.9850	**5532.1847** **5532.1847** **6.88e-11**	5532.1847 5532.1859 0.0045	5532.1847 5532.1847 2.49e-05
Cancer	Best Average Std	2967.2399 2968.5959 0. 8094	**2964.3869** **2964.3869** **7.31e-13**	2964.3869 2964.3869 9.37e-09	2964.3869 2964.3869 6.49e-09

**Table 18:** Comparison of the accuracy parameter for the results of the CSS and CSSRank algorithms.

Dataset	Criteria	CSS	CSSRank		
			Roulette wheel	Tournament	Random
Iris	Average Std	0.9131 0.0107	**0.9213** **0.0088**	0.9207 0.0107	0.9209 0.0095
Glass	Average Std	0.6935 0.0321	**0.7198** **0.0104**	0.7048 0.0319	0.7034 0.0242
Wine	Average Std	0.7335 0.0181	**0.7513** **0.0117**	0.7500 0.0132	0.7509 0.0145
Vowel	Average Std	0.8253 0.0127	**0.8472** **0.0140**	0.8383 0.0145	0.8410 0.0146
CMC	Average Std	0.5260 0.0267	0.5414 0.0234	**0.5434** **0.0267**	0.5378 0.0243
Cancer	Average Std	0.9425 0.0085	0.9680 0.0033	**0.9681** **0.0038**	0.9676 0.0030

To further validate the competitiveness of CSSRank, its performance was compared with eight well-established metaheuristic algorithms commonly cited in the clustering literature. The results, taken directly from^42,43^, are summarized in Tables 19 and 20, which include K-means, PSO, ACO, ABC, DE, GA, BB-BC, and BAT algorithms. These tables also include results from CSS and CSSRank under all three selection strategies for a comprehensive comparison. Table 19 presents the results based on average intra-cluster distance. CSSRank achieved lower intra-cluster distances than all compared algorithms across most datasets. The only exceptions were the Iris and Wine datasets, where K-means and DE, respectively, achieved the best performance. Table 20 shows the accuracy comparisons. CSSRank outperformed all other algorithms across the majority of datasets using all three selection methods. The only notable exception was the Vowel dataset, where the GA algorithm achieved accuracy nearly identical to that of CSSRank with roulette wheel selection.

**Table 19:** Comparison of the average intra-cluster for the results of CSSRank and other algorithms.

Dataset	K-means	PSO	ACO	ABC	DE	GA	BB-BC	BAT	CSS	CSSRank		
										Roulette Wheel	Tournament	Random
Iris	**9.20E+01**	9.86E+01	1.01E+02	1.08E+02	1.21E+02	1.25E+02	9.68E+01	1.15E+02	9.67 E+01	9.66 E+01	9.66 E+01	9.66 E+01
Glass	3.79E+02	2.76E+02	2.19E+02	3.29E+02	3.62E+02	2.82E+02	6.64E+02	3.75E+02	2.37 E+02	**2.15 E+02**	2.17 E+02	2.19 E+02
Wine	1.81E+04	1.64E+04	1.62E+04	1.69E+04	**1.58E+04**	1.65E+04	1.67E+04	1.71E+04	1.63 E+04	1.62 E+04	1.62 E+04	1.62 E+04
Vowel	1.60E+05	1.58E+05	1.89E+05	1.70E+05	1.81E+05	1.59E+05	1.94E+05	1.96E+05	1.51 E+05	**1.49 E+05**	1.49 E+05	1.49 E+05
CMC	5.59E+03	5.85E+03	5.83E+03	5.94E+03	5.95E+03	5.76E+03	5.71E+03	5.79E+03	5.69 E+03	**5.53 E+03**	5.53 E+03	5.53 E+03
Cancer	1.93E+04	4.26E+03	3.37E+03	3.50E+03	3.73E+03	3.00E+03	2.96E+03	3.06E+03	2.96 E+03	**2.96 E+03**	2.96 E+03	2.96 E+03

**Table 20:** Comparison of the average accuracy for the results of CSSRank and other algorithms.

Dataset	K-means	PSO	ACO	ABC	DE	GA	BB-BC	BAT	CSS	CSSRank		
										Roulette wheel	Tournament	Random
Iris	82.33	84.13	72.87	89.03	88.37	78.34	83.25	90.5	91.31	**92.13**	92.07	92.09
Glass	51.87	53.73	37.36	48.43	48.48	48.97	55.53	48.76	69.35	**71.98**	70.48	70.34
Wine	67.53	67.94	59.21	70.34	71.1	65.73	66.43	65.48	73.35	**75.13**	75	75.09
Vowel	51.16	84.04	51.69	56.31	53.41	**84.7**	84.32	57.21	82.53	**84.72**	83.83	84.10
CMC	39.69	44.1	36.89	40.06	39.58	43.3	44.67	42.62	52.60	54.14	**54.34**	53.78
Cancer	96.5	93.62	77.92	95.01	94.68	55.73	57.04	89.21	94.25	96.80	**96.81**	96.76

In conclusion, CSSRank demonstrated superior clustering performance in terms of both intra-cluster compactness and classification accuracy. The roulette wheel selection method consistently yielded the best results among the selection strategies, confirming its effectiveness within the CSSRank framework.

## Application of CSSRank to optimal operation of multi-reservoir system

Reservoir operation optimization (ROO) poses a significant challenge due to its complex nature, involving dynamically constrained nonlinear problems with multiple decision variables and objectives, as well as inherent risks and uncertainties. To address this, we propose three reservoir operation problems:

- Two hypothetical benchmark problems of multi-reservoir operation, focusing on maximizing hydropower generation benefits. These include a four-reservoir and a ten-reservoir system.
- A realistic three-reservoir system situated in the Maharloo-Bakhtegan basin in central Iran, aimed at minimizing water supply deficits over a 60-month period.

**Fig. 14:**
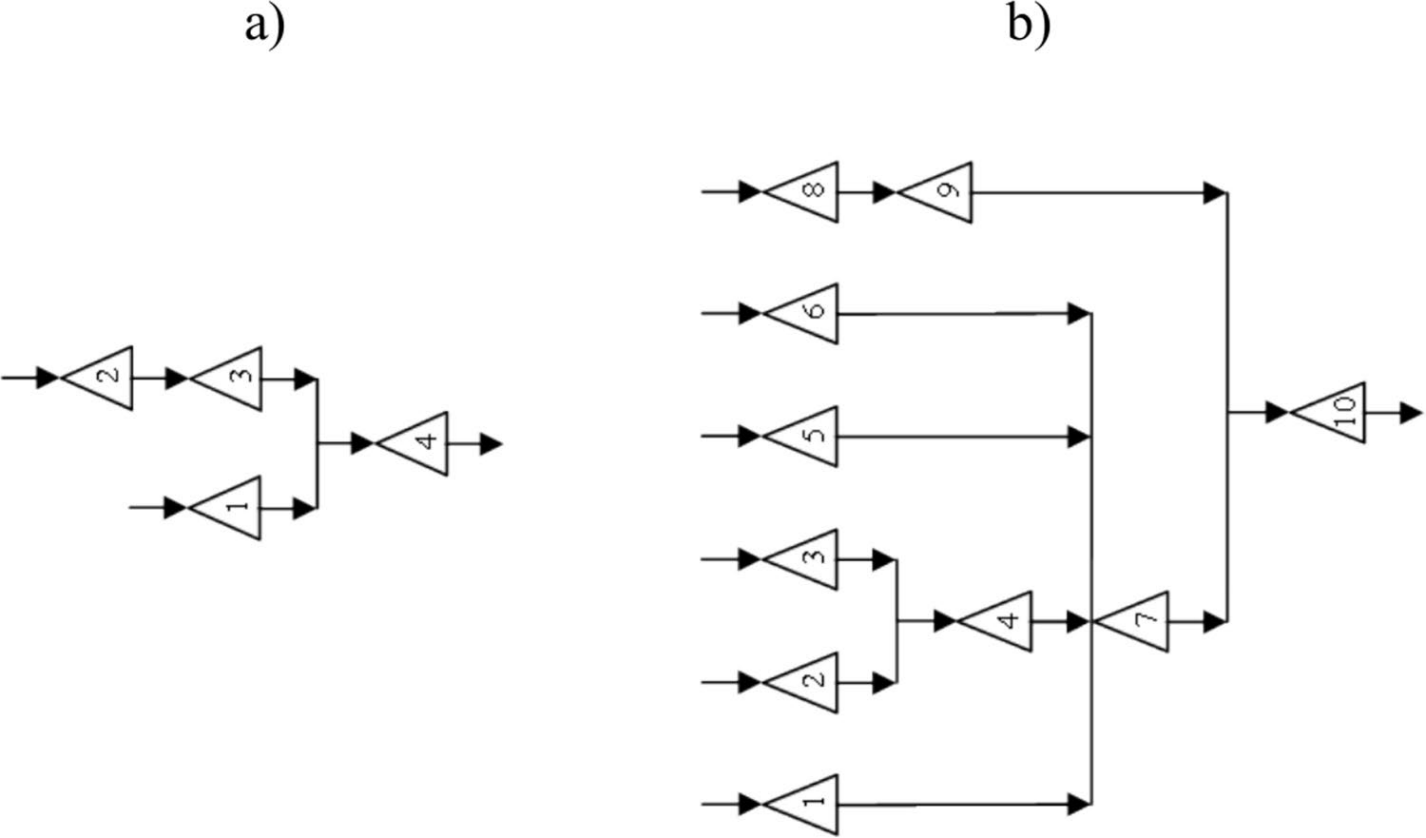
Schematic representation of the (**a**) four-reservoir and (**b**) ten-reservoir problems.

**Fig. 15:**
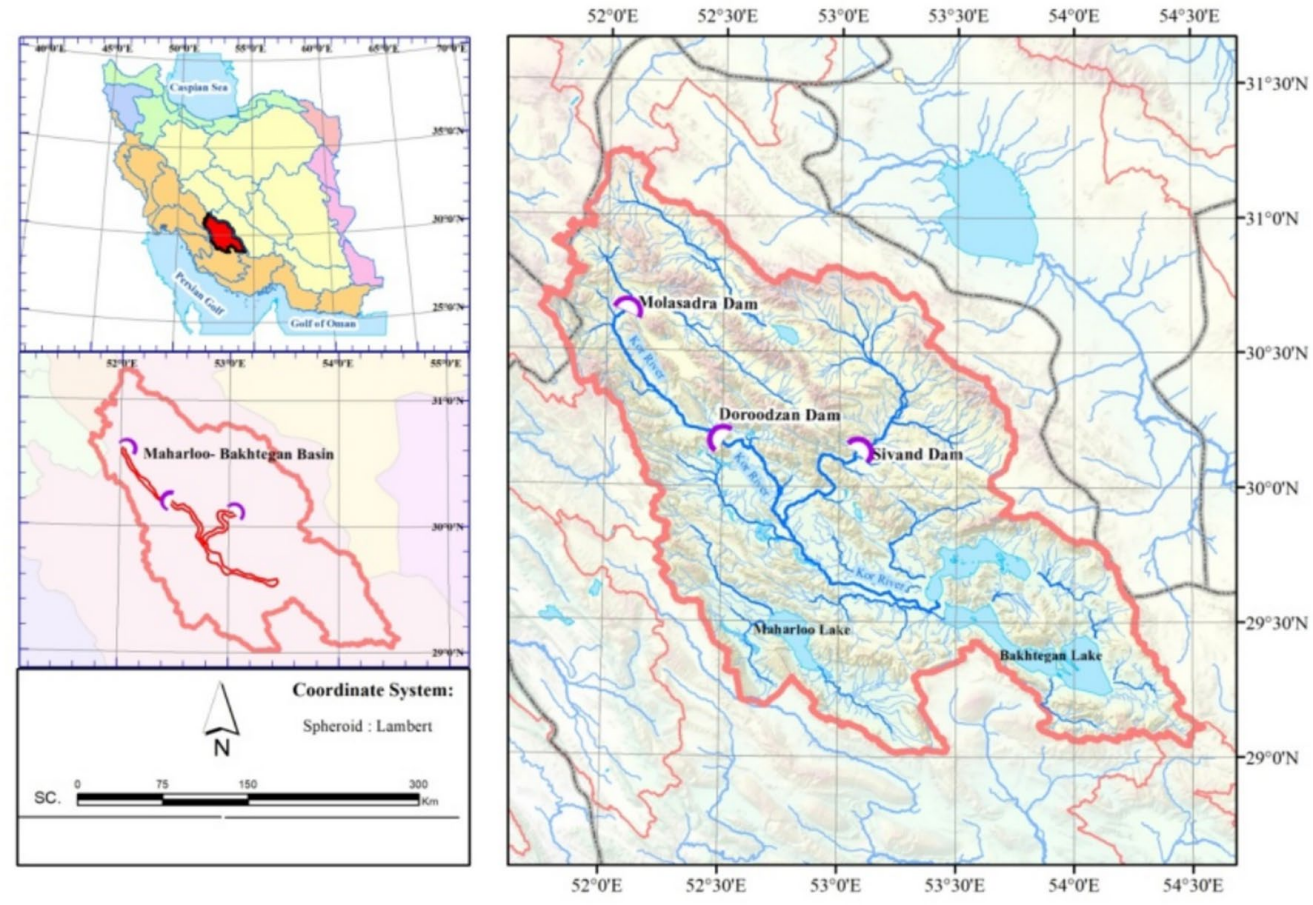
Location of Maharloo-Bakhtegan basin dams.

The hypothetical multi-reservoir system’s schematic is illustrated in Fig. 14, while Fig. 15 and Table 21 provide details on the dams’ locations and characteristics. The overarching objective function seeks to maximize benefits (or minimize negative benefits) for the four- and ten-reservoir systems and minimize the total deficit for the three-reservoir system as:

18 $$Minimize\quad F\left( {Re} \right) = a \times \sum\limits_{i = 1}^{N} {\sum\limits_{t = 1}^{T} {\left( {\frac{{Re_{i,t} - De_{i,t} }}{{De_{\max i} }}} \right)^{2} + } } \left( {a - 1} \right) \times \sum\limits_{i = 1}^{N} {\sum\limits_{t = 1}^{T} {b_{i,t} \times Re_{i,t} } }$$

where *i* is the counter of the reservoir number; *N* is the total number of reservoirs; *t* is the counter of operation period; *T* is the total of operation period; $Re_{i,t}$ is the release of reservoir *i* in period *t*; $De_{i,t}$ is the demand of reservoir *i* in period *t*; $De_{\max i}$ is the maximum downstream demand of reservoir *i* in period *t*; $b_{i,t}$ is the benefit per unit of the release of reservoir *i* in period *t*; and *a* is a coefficient that can be either 1 (for the realistic three-reservoir system) or 0 (for the four- and ten-reservoir systems).

**Table 21:** Main characteristics of the three-reservoir system.

Characteristics	Molasadra dam	Doroodzan dam	Sivand dam
Type of dam	Earth dam	Earth dam	Earth dam
The first year of operation	2006	1974	2007
Height from the foundation (m)	72	60	57
Crest length	630	710	600
Total storage capacity (MCM)	440	960	150
Irrigated land area (Km^2^)	100	1120	107

The operation of each reservoir adheres to the continuity constraint, which is formulated as follows for reservoir *i* over operating period *t*:

19 $$S_{i,t + 1} = S_{i,t} + I_{i,t} - Loss_{i,t} - M.Re_{i,t} \quad \forall t = 1,...,T\quad \& \quad i = 1,...,N$$

20 $$Loss_{i,t} = A_{i,t} \times Ev_{i,t} \quad \forall t = 1,...,T\quad \& \quad i = 1,...,N$$

where, $S_{i,t}$ is the storage of reservoir *i* at the start of period *t*; $I_{i,t}$ is the inflow to reservoir *i* in the period *t*; $Loss_{i,t}$ is the loss from reservoir *i* in the period *t*; *M* is a *N×N* matrix of indices of reservoir connectivity of water releases and inflow; $A_{i,t}$ is the area of reservoir *i* in the period *t* that is formulated in terms of reservoir storage; and $Ev_{i,t}$ is the evaporation of reservoir *i*. The optimization procedure is subject to the following constraints:

21 $$Re_{i}^{\min } \le Re_{i,t} \le Re_{i}^{\max } \quad \forall t = 1,...,T\quad \& \quad i = 1,...,N$$

22 $$S_{i}^{\min } \le S_{i,t} \le S_{i}^{\max } \quad \forall t = 1,...,T\quad \& \quad i = 1,...,N$$

23 $$S_{i,T + 1} = S_{i,1} \quad \forall i = 1,...,N$$

where $Re_{i}^{\min }$ is the minimum release of reservoir *i*; $Re_{i}^{\max }$ is the maximum release of reservoir *i* $S_{i}^{\min }$ is the minimum storage of reservoir *i*; and $S_{i}^{\max }$ is the maximum storage of reservoir *i* in the period *t*. Spillage is calculated when reservoir storage exceeds its maximum storage as follows:

24 $$Sp_{i,t} = S_{i,t} - S_{i}^{\max } \Rightarrow \;S_{i,t} = S_{i}^{\max } \quad \forall t = 1,...,T\quad \& \quad i = 1,...,N$$

where $Sp_{i,t}$ is the spillage of reservoir *i* in the period *t*. The penalty functions must be added when the reservoir storage does not meet the constraints. $P1_{i,t}$, $P2_{i,t}$ and $P3_{i,t}$ are the penalties costs related to the storage of reservoir *i* in the period *t* that are respectively defined as:

25 $$P1_{i,t} = \left\{ \begin{gathered} C_{1} \left( {S_{i}^{\min } - S_{i,t} } \right)^{2} \quad :if\quad S_{i,t} < S_{i}^{\min } \hfill \\ 0\quad \quad \quad \quad \quad \quad \quad \quad \quad :otherwise \hfill \\ \end{gathered} \right.\quad for\;t = 1,...,T\,\& \,i = 1,...,N$$

26 $$P2_{i,t} = \left\{ \begin{gathered} C_{2} \left( {S_{i,t} - S_{i}^{\max } } \right)^{2} \quad :if\quad S_{i,t}> S_{i}^{\max } \hfill \\ 0\quad \quad \quad \quad \quad \quad \quad \quad \quad :otherwise \hfill \\ \end{gathered} \right.\quad for\;t = 1,...,T\,\& \,i = 1,...,N$$

27 $$P3_{i} = C_{3} \left( {S_{i,T + 1} - S_{i,1} } \right)^{2} \quad :if\quad S_{i,T + 1} \ne S_{i,1} \quad for\quad i = 1,...,N$$

where *C*_1_, *C*_2_, and *C*_3_ are the penalty coefficients for the violation of penalties on maximum storage, minimum storage, and inequality penalties on initial and target storage, respectively. Finally, the penalized objective function can be expressed as follows:

28 $$\begin{gathered} Minimize\quad F\left( {Re} \right) = a \times \sum\limits_{i = 1}^{N} {\sum\limits_{t = 1}^{T} {\left( {\frac{{Re_{i,t} - De_{i,t} }}{{De_{\max i} }}} \right)^{2} + } } \left( {a - 1} \right) \times \sum\limits_{i = 1}^{N} {\sum\limits_{t = 1}^{T} {b_{i,t} \times Re_{i,t} } } \hfill \\ \quad \quad \quad \quad \quad \quad \quad \quad \quad + \sum\limits_{i = 1}^{N} {\sum\limits_{t = 1}^{T} {\left( {P1_{i,t} + P2_{i,t} + P3_{i,t} } \right)} } \hfill \\ \end{gathered}$$

### Four-reservoir problem results

The four-reservoir problem serves as a benchmark for ROO, initially tackled by Chow and Cortes-Rivera^44^ and Murray and Yakowitz^45^. This problem involves a multi-reservoir system comprising both series and parallel reservoirs, operating over 12-month periods to meet hydropower generation and irrigation demands, as shown in Fig. 14(a). The input data necessary for the reservoir operation optimization (ROO) problems include various parameters such as inflows, initial storages, reservoir connectivity matrix, reservoir storages, release constraints, and benefit functions. These details are extensively discussed and documented in Ref^45^..

**Fig. 16:**
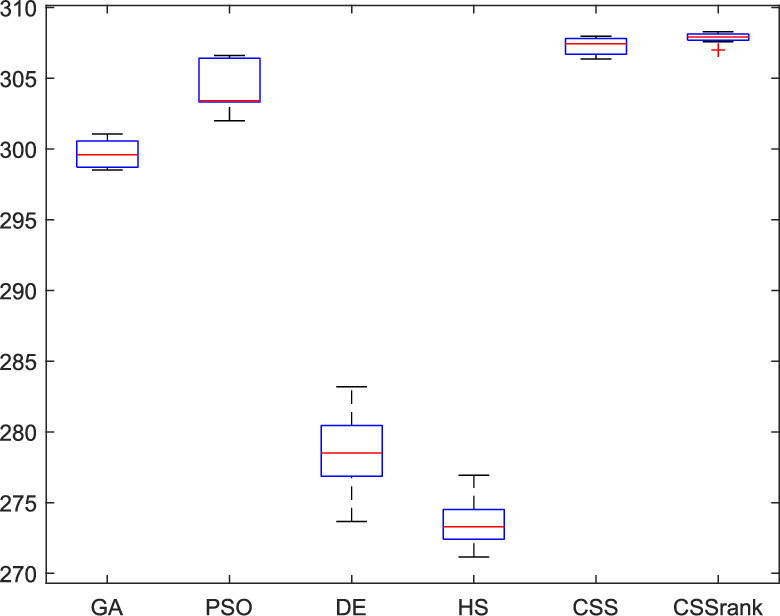
Results of different runs in four-reservoir problem.

The results from 30 runs of all selected algorithms, each with 500,000 function evaluations, are depicted in Fig. 16. Table 22 shows that CSSRank achieved a solution closer to the global optimum, with its best solution equaling the global solution of 308.29. Notably, CSSRank exhibited a mere 0.149% difference from the global optimal solution on average. Moreover, CSSRank ‘s worst solution outperformed the average solutions obtained by other algorithms, except for the original CSS. Additionally, CSSRank demonstrated lower standard deviation and coefficient of variation compared to other algorithms, underscoring its accuracy and ability to approach near-optimal global solutions for the four-reservoir problem. Conversely, the original CSS outperformed other algorithms, yielding solutions of 307.97, 307.31, and 306.36 as the best, average, and worst solutions, respectively.

**Table 22:** Summary of results of different runs in four-reservoir problem.

	GA	PSO	DE	HS	CSS	CSSRank
Best	301.06	306.61	283.19	276.93	307.97	308.29
Worst	298.51	302.00	273.67	271.16	306.36	306.99
Average	299.63	304.37	278.42	273.60	307.31	307.83
Standard deviation	0.9707	1.78	2.96	1.7507	0.5619	0.3744
Coefficient of variation	0.0032	0.0058	0.0106	0.0064	0.0018	0.0012
Best solution*	308.09	308.22	305.99	303.48	308.29	308.29

^*^after 10,000,000 function evaluations.

**Fig. 17:**
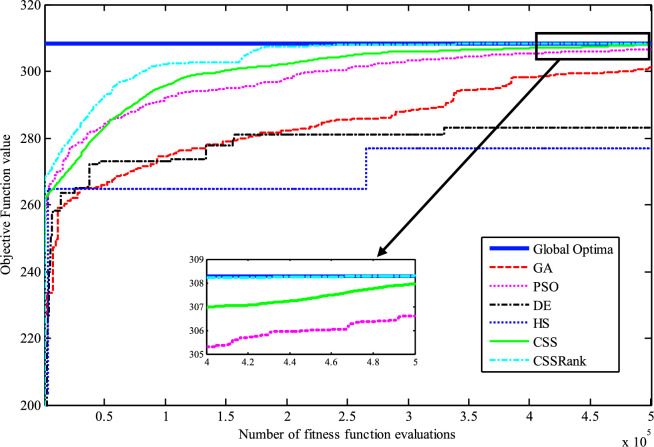
Objective value evaluation of four-reservoir operation by proposed algorithms (the best of runs).

**Fig. 18:**
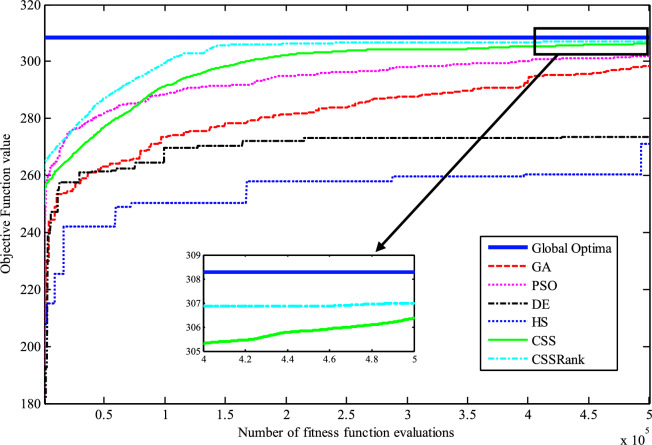
Objective value evaluation of four-reservoir operation by proposed algorithms (the worst of runs).

**Fig. 19:**
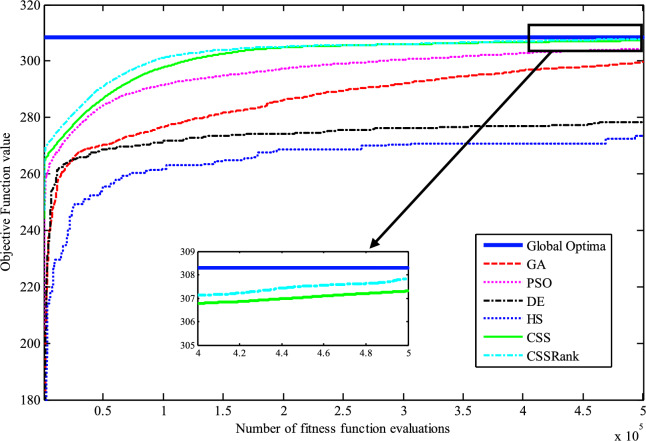
Objective value evaluation of four-reservoir operation by proposed algorithms (averaged over different runs).

**Fig. 20:**
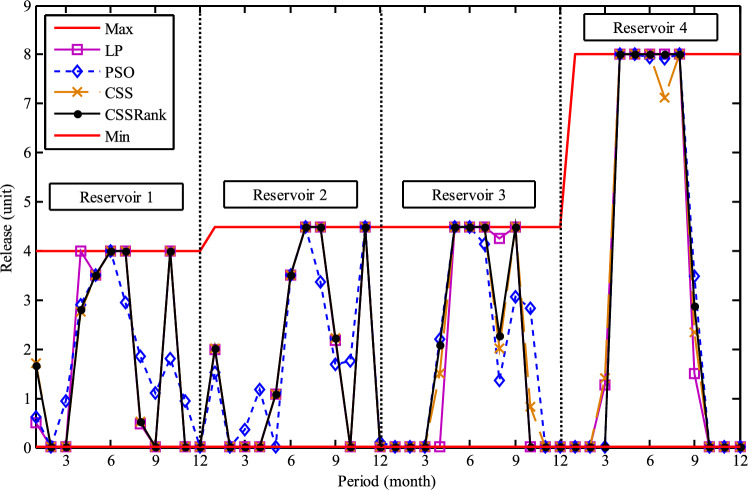
Comparison of the best results of PSO, CSS, and LP model for reservoir releases (four-reservoir problem).

**Fig. 21:**
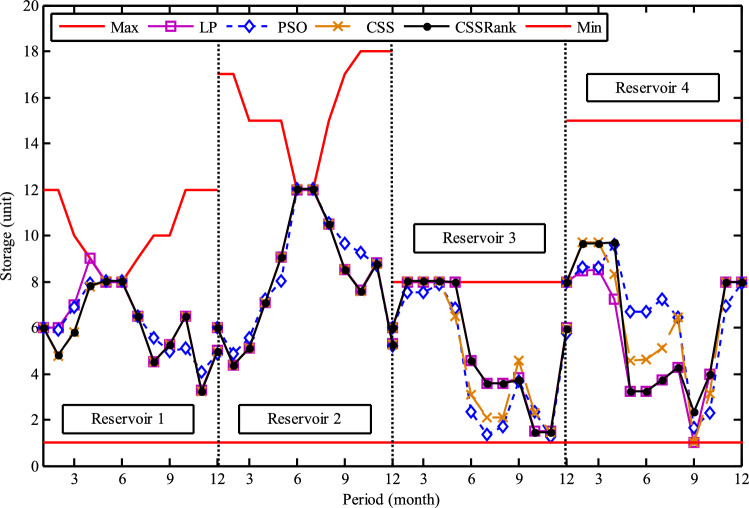
Comparison of the best results of PSO, CSS, and LP models for reservoir storages (four-reservoir problem).

In the final row of Table 22, the results from an extended simulation run are presented, indicating that all algorithms achieved very good convergence to near-optimal solutions after approximately 10^7 function evaluations. Notably, both CSS and its enhanced version, CSSRank, successfully reached the global solution. Figs. 17,18,19 depict convergence rate plots against the number of function evaluations for the best, worst, and average solutions obtained across 30 runs for all proposed algorithms. These results illustrate that CSS and CSSRank exhibit superior and faster convergence compared to other algorithms toward achieving the optimal solution. Figs. 20 and 21 showcase the monthly reservoir release and reservoir storage patterns of the best solution for the four-reservoir system for the three top-performing algorithms.

### Ten-reservoir problem results

**Fig. 22:**
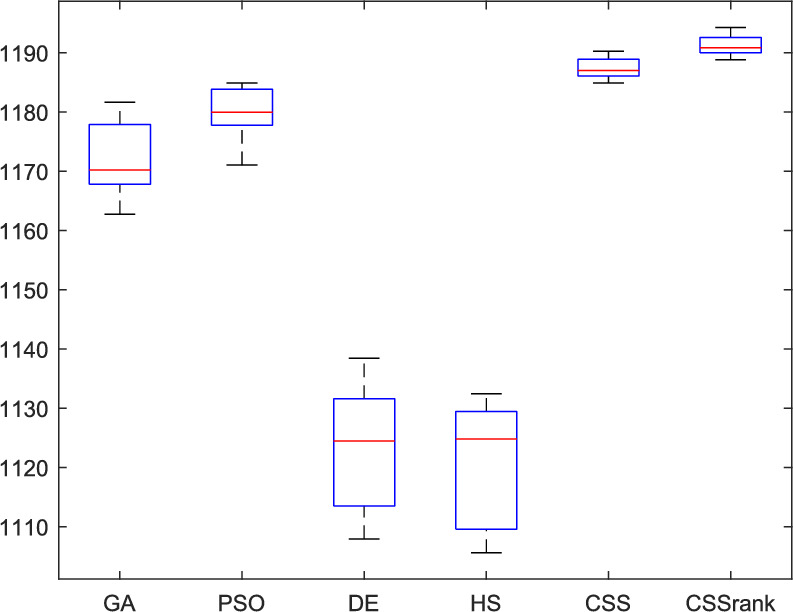
Results of different runs in ten-reservoir problem.

**Fig. 23:**
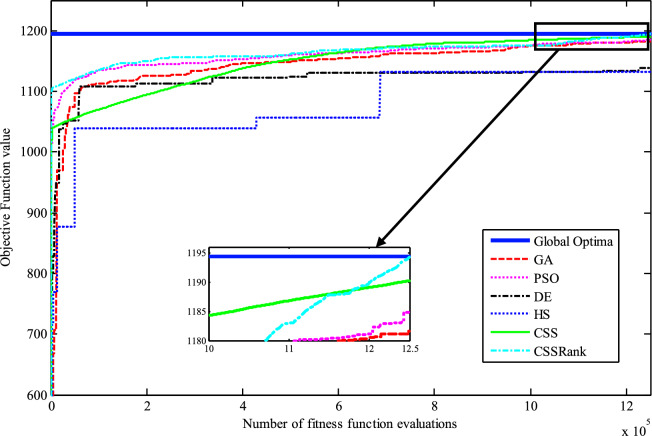
Objective value evaluation of ten-reservoir operation by proposed algorithms (the best of runs).

**Fig. 24:**
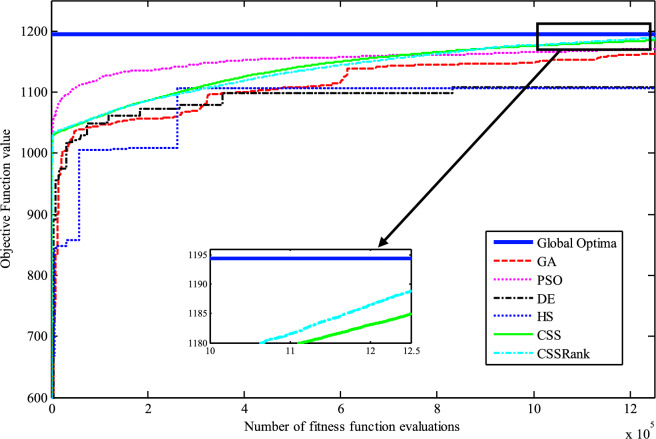
Objective value evaluation of ten-reservoir operation by proposed algorithms (the worst of runs).

**Fig. 25:**
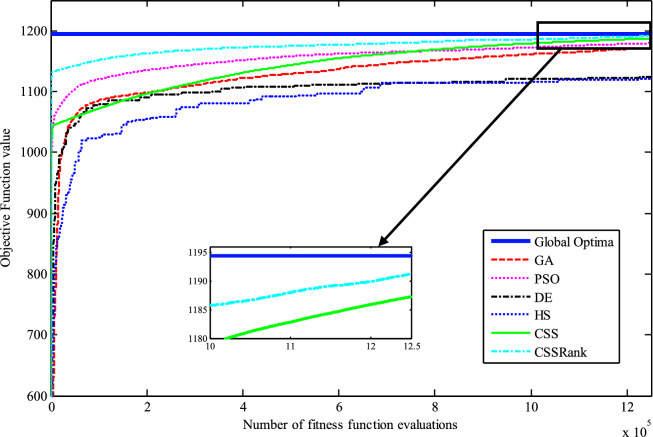
Objective value evaluation of ten-reservoir operation by proposed algorithms (averaged over different runs).

**Fig. 26:**
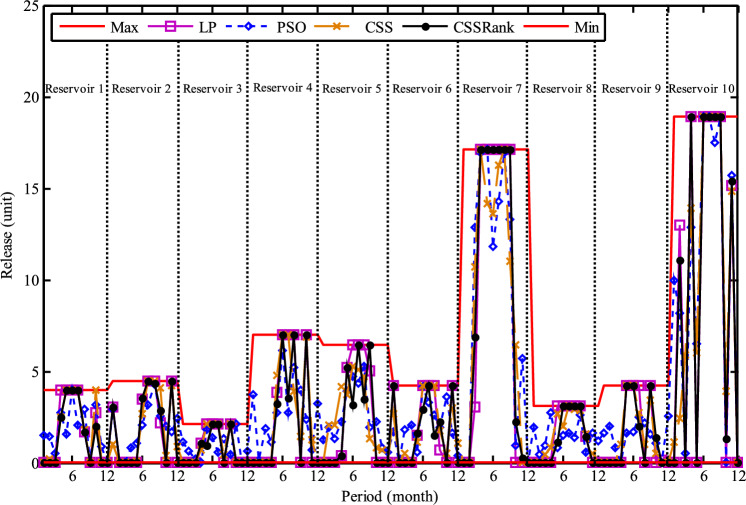
Comparison of the best results of PSO, CSS, and LP model for reservoir releases (ten-reservoir problem).

**Fig. 27:**
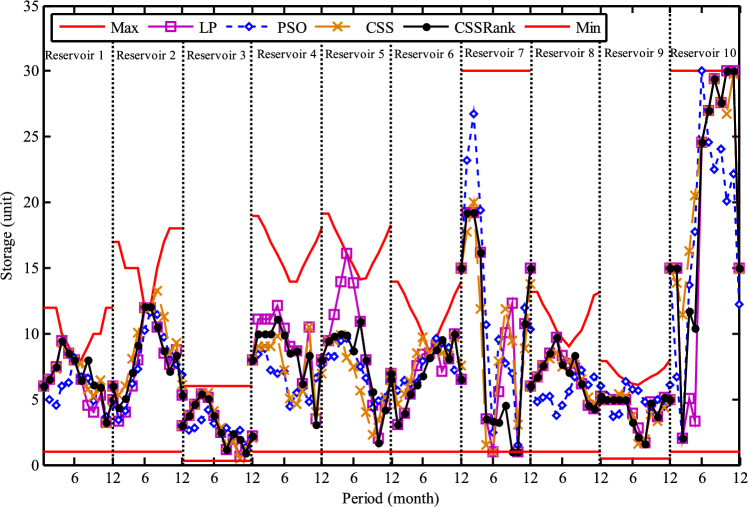
Comparison of the best result of PSO, CSS, and LP model for reservoir storages (ten-reservoir problem).

The ten-reservoir problem was initially introduced by Murray and Yakowitz^45^. For our study, 30 runs of the proposed algorithms were conducted with a maximum of 1,250,000 function evaluations. The results, depicted in Fig. 22 and summarized in Table 23, reveal relative errors for the average objective function values of approximately 1.93%, 1.25%, 5.96%, 6.10%, 0.6%, and 0.3% for GA, PSO, DE, HS, CSS, and CSSRank, respectively, compared to the global solution. Notably, CSSRank demonstrates a suitable performance in reaching the global optimal solution, with standard deviation and coefficient of variation values close to those of CSS. In contrast, PSO exhibits a significantly larger standard deviation of the objective function value, approximately 2.5 times that of CSSRank in different runs. The best, average, and worst values of the objective function achieved by CSSRank are 99.98%, 99.74%, and 99.53% of the global optimal solution, respectively. In the extended run with 5×10^7 function evaluations, the CSSRank algorithm converges to 1194.23, closely approaching the global optimal solution compared to other algorithms, as presented in Table 23. Convergence rates of the best, worst, and average solutions over the 30 runs are visualized in Figs. 23,24,25 illustrating the superior performance of CSSRank over other methods. Furthermore, monthly releases and storages from different reservoirs, along with allowable ranges, are presented in Figs. 26 and 27 to provide additional insight into the optimization process.

**Table 23:** Summary of results for different runs in ten-reservoir problem.

	GA	PSO	DE	HS	CSS	CSSRank
Best	1181.62	1184.87	1138.41	1132.41	1190.25	1194.23
Worst	1162.73	1171.02	1107.93	1105.58	1184.89	1188.79
Average	1171.42	1179.53	1123.30	1121.53	1187.26	1191.29
Standard deviation	6.2079	4.5296	9.8891	10.0542	1.8219	1.8125
Coefficient of variation	0.0053	0.0038	0.0088	0.0090	0.0015	0.0015
Best solution*	1191.82	1192.56	1177.41	1180.11	1194.09	1194.23

^*^after 50,000,000 function evaluations.

### Statistical study

The statistical analysis conducted on the performance of various optimization algorithms for reservoir problems revealed significant differences among the methods, as outlined in Table 24. The Friedman ranks test indicated a notable distinction between the developed algorithm and others for both the four-reservoir and ten-reservoir problems, highlighting its efficacy in finding optimal solutions, particularly for the challenging ten-reservoir problem. This finding was further supported by the Friedman-aligned ranks test, reinforcing the superiority of the proposed algorithm across both problem instances. Additionally, the Quade test underscored the CSSRank algorithm’s superior performance, consistently ranking it highest among all optimization algorithms for both scenarios. Furthermore, the Wilcoxon signed-rank test demonstrated a statistically significant improvement in CSSRank performance compared to other algorithms, confirming its effectiveness in reservoir optimization tasks. These results collectively indicate CSSRank’s potency as a robust optimization algorithm for enhancing reservoir management practices, ultimately leading to increased efficiency and effectiveness in reservoir operations.

**Table 24:** Statistical results.

**Method**	***Friedman ranks***			
	**4-reservoir problem**		**10-reservoir problem**	
	***R***	**Rank**	***R***	**Rank**
**GA**	52.73	4	51.63	4
**PSO**	35.83	3	37.97	3
**DE**	69.98	5	73.97	5
**HS**	80.52	6	76.53	6
**CSS**	18.36	2	22.5	2
**CSSRank**	**14.06**	**1**	**8.9**	**1**
**Method**	***Friedman-aligned ranks***			
	**4-reservoir problem**		**10-reservoir problem**	
	***R***	**Rank**	***R***	**Rank**
**GA**	4.00	4	3.96	4
**PSO**	2.90	3	3.03	3
**DE**	5.13	5	5.33	5
**HS**	5.86	6	5.66	6
**CSS**	1.86	2	1.96	2
**CSSRank**	**1.23**	**1**	**1.03**	**1**
**Method**	***Quade test***			
	**4-reservoir problem**		**10-reservoir problem**	
	***R***	**Rank**	***R***	**Rank**
**GA**	1.01	4	1.01	4
**PSO**	0.73	3	0.76	3
**DE**	1.32	5	1.35	5
**HS**	1.48	6	1.44	6
**CSS**	0.46	2	0.49	2
**CSSRank**	**0.33**	**1**	**0.27**	**1**
**Method**	***Wilcoxon signed-rank*** ***test***			
	**4-reservoir problem**		**10-reservoir problem**	
	**CSSRank**		**CSSRank**	
**GA**	+		+	
**PSO**	+		+	
**DE**	+		+	
**HS**	+		+	
**CSS**	+		+	

### Realistic three-reservoir results

The realistic multi-reservoir system, illustrated in Fig. 15, comprises three reservoirs. The objective function aims to minimize the total water supply deficit over a period of 5 years or 60 operating periods (months), as defined in Eq. (23) with *a* = 1. Decision variables are optimized monthly releases from each reservoir, while the state variables represent the storage volumes of the reservoirs. With 60 time periods and three decisions in each period, the total number of decision variables in this three-reservoir system equals 180, corresponding to the dimensions of the problem. Constraints posed on reservoir releases, *Re*_*i,t*_, and reservoir storages, *S*_*i,t*_, for each reservoir are as follows:

29 $$60.1\le {S}_{1,t}\le 440\hspace{1em}\forall t=1,...,T$$

30 $$170\le {S}_{2,t}\le 960.85\hspace{1em}\forall t=1,...,T$$

31 $$12\le {S}_{2,t}\le 150\hspace{1em}\forall t=1,...,T$$

where $S_{1,t}$, $S_{2,t}$ and $S_{3,t}$ denote the storage of the Molasadra reservoir, Doroodzan reservoir, and Sivand reservoir in the period *t*, respectively.

Using a population size of 200 and a maximum of 1000 iterations, we compared the results of CSS and CSSRank with those of other methods in Table 25. CSSRank achieved the optimal value for the operation of the realistic three-reservoir system, outperforming the other algorithms. CSSRank supplied 99.18%, 98.18%, and 91.78% of the total downstream demands of the Molasadra, Doroodzan, and Sivand dams, respectively.

**Table 25:** Results of different runs for three-reservoir problem.

Algorithm	Optimal value	Molasadra deficit (MCM)	Doroodzan deficit (MCM)	Sivand deficit (MCM)
GA	0.246	29.32	224.76	3.56
PSO	0.061	3.71	78.85	2.02
DE	0.080	13.93	74.79	2.25
HS	0.245	24.70	235.68	2.86
CSS	0.060	4.36	51.55	2.11
CSSRank	0.055	2.15	33.73	2.03

**Fig. 28:**
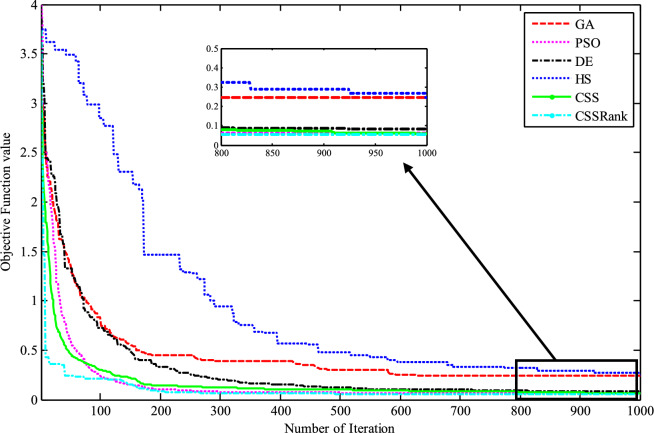
Objective value evaluation of realistic three-reservoir operation by proposed algorithms.

**Fig. 29:**
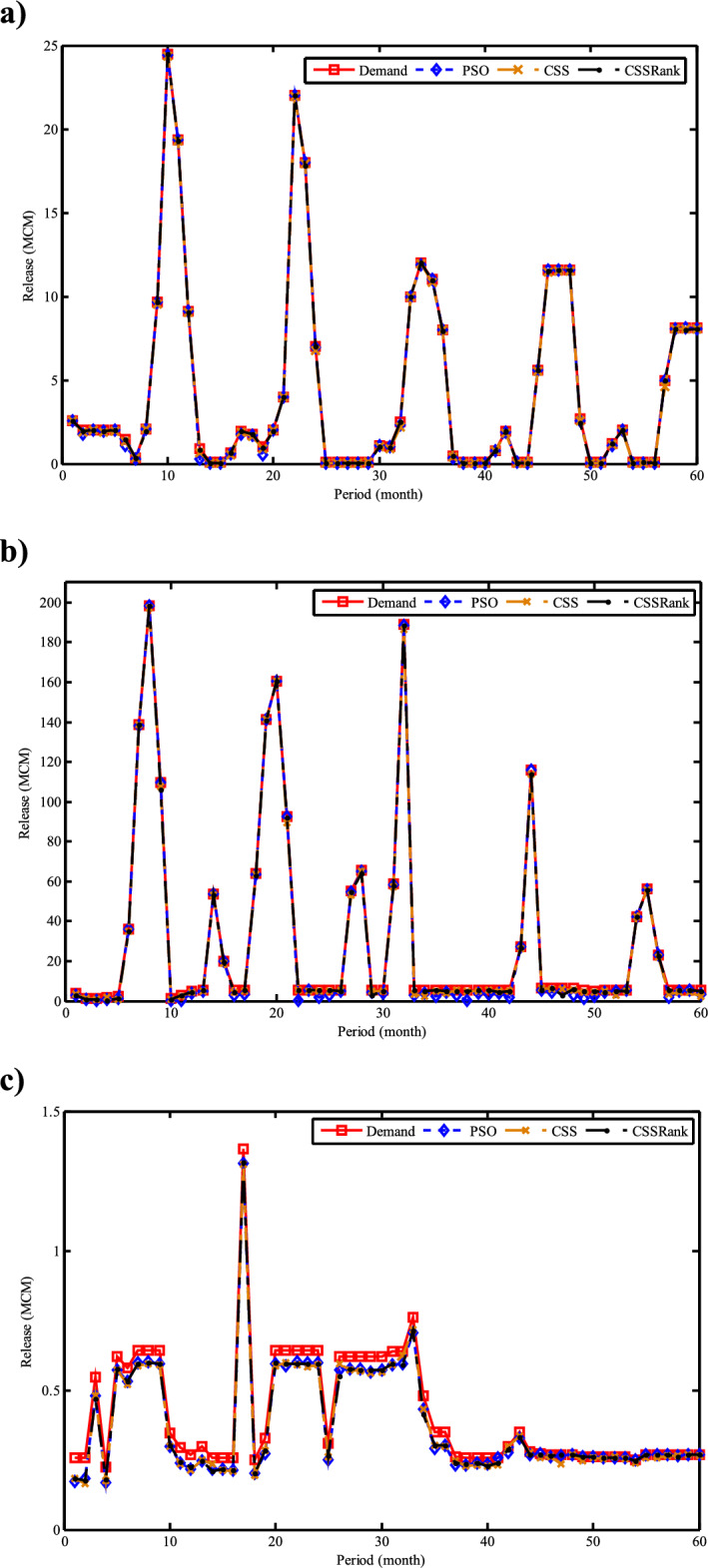
Monthly optimal releases from (**a**) Molasadra, (**b**) Doroodzan, and (**c**) Sivand reservoirs.

**Fig. 30:**
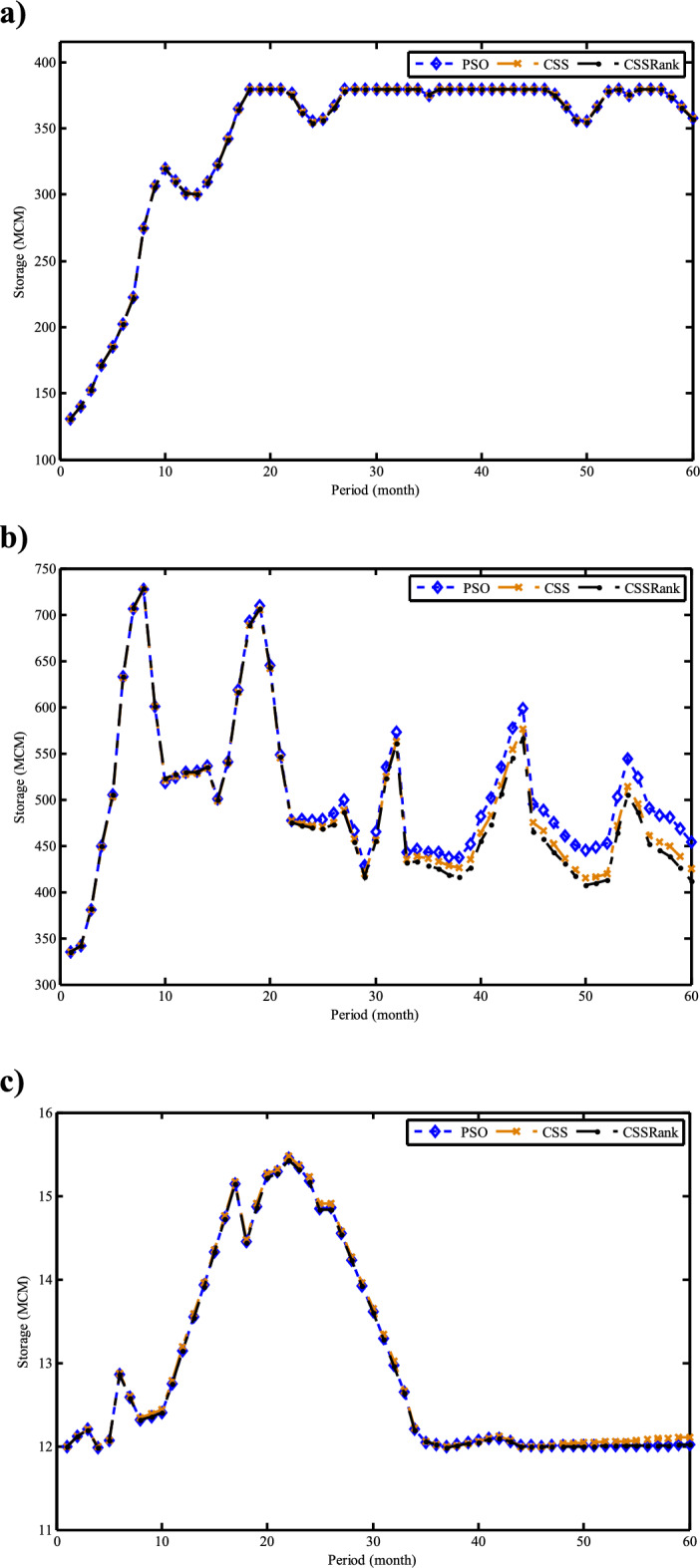
Monthly optimal water storage of (**a**) Molasadra, (**b**) Doroodzan, and (**c**) Sivand reservoirs.

**Fig. 31:**
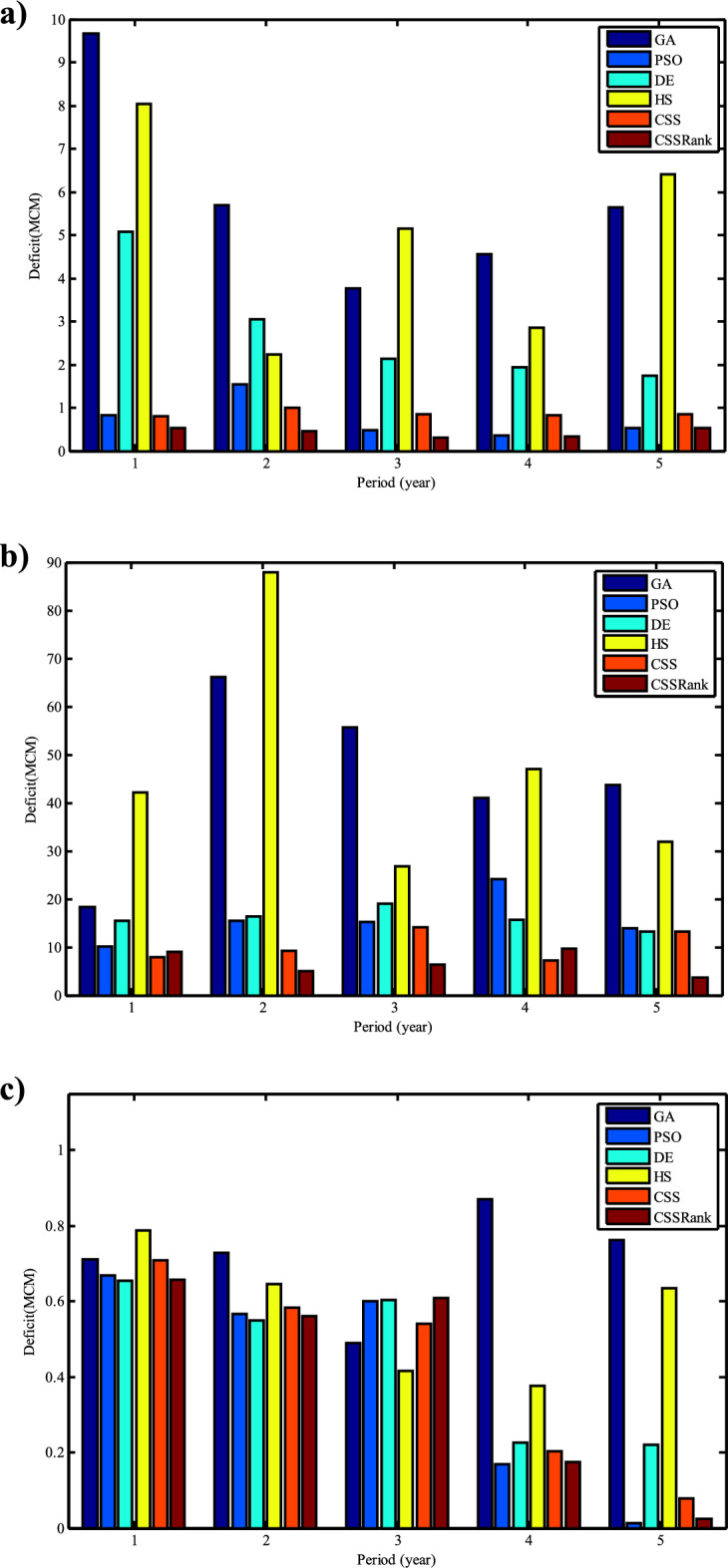
Annual deficits in (**a**) Molasadra, (**b**) Doroodzan, and (**c**) Sivand reservoirs.

Fig. 28 depicts the convergence rate of the applied algorithms in reaching the optimum value for the operation of the realistic three-reservoir system. CSSRank rapidly converged compared to the other algorithms, requiring fewer iterations to achieve the optimal value. Fig. 29 displays the release amounts obtained from the three best algorithms against the downstream demands during the study period. CSSRank reliably supplied the downstream demand of the three reservoirs. Fig. 30 illustrates the monthly storage of Molasadra, Doroodzan, and Sivand reservoirs. The reservoir volumes calculated by the applied algorithms are within the allowable range of reservoir storage. Fig. 31 presents the annual deficits of the reservoirs obtained by the applied algorithms. CSSRank yielded the lowest total deficit during the study period, indicating its superior performance in minimizing the total water supply deficit.

## Discussion and conclusion

This study introduces CSSRank, a structurally enhanced version of the CSS algorithm, which addresses specific limitations of the original method through two core innovations designed to improve performance on complex optimization problems: the first enhancement is the introduction of a rank-based reduction selection strategy, which dynamically reduces the number of CPs involved in force calculations during each iteration. Unlike the original CSS algorithm, which computes the resultant electric force using all CPs, CSSRank selectively focuses on a subset of top-ranked particles. This significantly reduces computational overhead while sharpening the algorithm’s exploitation capabilities, especially in later iterations. Moreover, by supporting multiple selection techniques, such as Tournament Selection, Roulette Wheel Selection, and Random Selection, CSSRank allows for flexible control over exploration pressure and maintains diversity in the early stages of the search. The second enhancement is a ranking-based mutation strategy, which perturbs elite particles based on their fitness rank and an adaptive mutation probability. Unlike traditional mutation approaches that apply fixed or random perturbations, this strategy targets a single dimension of high-quality solutions, introducing controlled variation without disrupting convergence. This helps CSSRank avoid premature convergence, especially in multimodal and deceptive landscapes, by maintaining a careful balance between exploration and exploitation throughout the search process.

These enhancements go beyond conventional parameter tuning or algorithmic hybridization and represent a fundamental redesign of the core mechanics of the CSS algorithm. By streamlining the computation of electric forces and incorporating rank-guided selection and mutation strategies, CSSRank achieves notable improvements in convergence speed, solution quality, and computational scalability. The effectiveness of these modifications is validated through extensive benchmark testing on the CEC 2014 and CEC 2024 suites, where CSSRank consistently matches or outperforms many state-of-the-art algorithms. These results confirm that CSSRank is not only a high-performing algorithm in theoretical test cases, but also a robust and adaptable framework well-suited for solving a diverse set of real-world optimization problems, including data clustering challenges and complex engineering design tasks.

To ensure a comprehensive evaluation, the selection of peer algorithms was guided by the distinct goals of each experimental context. For the benchmark comparisons, particularly those involving CEC 2014 and CEC 2024 test suites, we deliberately selected a diverse and competitive group of state-of-the-art metaheuristic algorithms, including recent competition winners and well-cited algorithms known for strong performance across various problem landscapes. This ensured that CSSRank was evaluated against the most rigorous and standardized benchmarks currently available. In contrast, for the real-world application studies, including clustering on UCI datasets and reservoir operation optimization, we focused on commonly accepted baseline methods that have been frequently employed and validated in the literature. These include widely used optimization methods in the field. The intent here was not to replicate the competitive rigor of the benchmark section, but to demonstrate the adaptability, stability, and practical utility of CSSRank when applied to realistic, domain-specific problems. This two-layered strategy, benchmarking for competitiveness and applications for versatility, ensures a balanced validation of the proposed method’s theoretical and practical strengths.

The results of benchmark mathematical examples demonstrate that the roulette wheel is the superior selection method. Also, numerical tests on the IEEE-CEC 2014 optimization test problems show the ability of CSSRank to find the global optimum of several types of problems with different dimensionalities and search landscapes. The proposed algorithm was also employed to address data clustering problems, where its performance was benchmarked against several established algorithms in the literature. Notably, CSSRank demonstrated its suitability for effectively tackling clustering problems. The utility of the CSSRank algorithm extends beyond benchmark functions and finds relevance in diverse engineering optimization problems, particularly those demanding a large number of variables, such as multi-reservoir operation optimization (ROO). The results show that the CSSRank algorithm demonstrated superior performance compared to other optimization algorithms, achieving minimal deficits in water supply for a realistic three-reservoir system, thus highlighting its effectiveness in difficult optimal operation research problems.

Comparing the computational complexity of the developed CSSRank algorithm with the original CSS highlights several key improvements. First, CSSRank introduces a rank-based reduction strategy, where only a selected subset of CPs, denoted as *sel*(*it*), are considered for force calculations, rather than all *N* particles as in standard CSS. This significantly reduces computational overhead. Since *sel*(*it*) satisfies the condition 0.5*N* < *sel*(*it*) < *N*, the number of pairwise interactions is notably decreased, resulting in a lower overall computational cost per iteration. Second, CSSRank replaces the predefined parameters CMCR and PAR with a success rate-based mechanism. This eliminates the need for manual parameter tuning while dynamically adapting the algorithm’s behavior, further reducing computational effort and simplifying the implementation. Third, instead of performing the full update process for each CP, which includes force computation, movement probability, velocity update, and position generation, CSSRank applies a dimension-wise mutation operator. This operator only perturbs one dimension of selected elite CPs, which is computationally cheaper than traditional update schemes. Together, these strategies contribute to reduced time complexity, improved efficiency, and enhanced scalability, making CSSRank suitable for a wide range of optimization problems, especially those with moderate to high dimensionality.

Looking ahead, this work not only advances the capabilities of the CSS algorithm but also lays a foundation for extending its core enhancements to other metaheuristic frameworks and problem domains. By introducing structural modifications, such as rank-based reduction and adaptive mutation strategies, CSSRank transcends the limitations of traditional CSS variants that remain constrained by the original algorithmic structure. This approach offers a versatile and adaptable optimization framework, with the potential to address a much broader spectrum of complex optimization challenges. Beyond its direct contributions, the methodology proposed in this study opens promising directions for future research, enabling innovation across diverse applications in metaheuristic optimization. In light of this, several potential avenues for future work are outlined as follows:

- Generalization for Other Methods: Extend the strategies developed for CSSRank to other metaheuristic algorithms to assess their applicability and effectiveness across a broader range of optimization techniques.
- Developing for High-Dimensional Problems: Adapting CSSRank for high-dimensional optimization problems to understand its scalability and effectiveness in scenarios with a large number of decision variables.
- Multi-Objective Optimization: Adapt provided strategies to handle multi-objective optimization problems.
- Hybridization with Machine Learning: Explore opportunities to hybridize CSSRank with machine learning algorithms to leverage the strengths of both approaches, potentially enhancing the algorithm’s ability to tackle complex optimization tasks with diverse data characteristics.
- New/Real-World Applications: Conduct empirical studies and validations on other new benchmark problems or real-world applications across various domains, such as neuro-heuristic analysis applied to video or pallet detection for vehicles, to assess the practical effectiveness and robustness of proposed techniques in solving practical optimization challenges.

## Data Availability

The datasets generated during the current study are available from the corresponding author on reasonable request.

## Abbreviations

CP: Charged Particle
${q}_{i}$: Uniform charge density
*fitbest*: The value of the objective function for the best CP
*fitworst*: The value of the objective function for the worst CP
*fit*(*i*): The value of the objective function for the *i*th CP
*N*: Total number of CPs
*r*_*ij*_: Separation distance between the *i*th and *j*th CPs
*X*_*bes*_: Position of the best current CP
*X*_*i*_: Position of the *i*th CP
*ε*: Small positive number
$x_{ij}^{(0)}$: Initial value of the *i*th variable for the *j*th CP
$x_{i,\min }$: Minimum allowable values for the *i*th variable
$x_{i,\max }$: Maximum allowable values for the *i*th variable
${P}_{ij}$: Probability of moving the *i*th-*j*th CPs
*F*_*j*_: Resultant force acting on the *j*th CP
${X}_{j,new}$: New position of the *j*th CP
${X}_{j,old}$: Old position of the *j*th CP
${V}_{j.new}$: New velocity of the *j*th CP
${V}_{j.old}$: Old velocity of the *j*th CP
*k*_*a*_: Acceleration coefficients
*k*_*v*_: Velocity coefficients
rand_*j*__1_, rand_*j*__2_: Two random numbers distributed in the range (0,1)
CM: Charged memory to save best CPs
*CMCR*: Probability of using CM to generate new solution
*PAR*: Probability of using neighboring of the best CP
*a*: Radius of the charged sphere
*Sel*_(__*i*__)_: Initial value of the selected percentage
*Sel*_(__*f*__)_: Final value of the selected percentage
*it*: Current iteration
*MaxIt*: Maximum number of iterations
*cpp*: Change probability parameter
*pm*: Mutation percentage
*RS*: Rate of success
